# Ester formation at the liquid–solid interface

**DOI:** 10.3762/bjnano.8.213

**Published:** 2017-10-12

**Authors:** Nguyen T. N. Ha, Thiruvancheril G Gopakumar, Nguyen D. C. Yen, Carola Mende, Lars Smykalla, Maik Schlesinger, Roy Buschbeck, Tobias Rüffer, Heinrich Lang, Michael Mehring, Michael Hietschold

**Affiliations:** 1 Solid Surfaces Analysis Group, Institute of Physics, Technische Universität Chemnitz, D-09107 Chemnitz, Germany; 2 Department of Chemistry, Indian Institute of Technology Kanpur, Kanpur 208016, India; 3 Inorganic Chemistry, Institute of Chemistry, Technische Universität Chemnitz, D-09107 Chemnitz, Germany,; 4 Coordination Chemistry, Institute of Chemistry, Technische Universität Chemnitz, D-09107 Chemnitz, Germany

**Keywords:** on-surface reaction, scanning tunneling microscopy, trimesic acid, undecan-1-ol

## Abstract

A chemical reaction (esterification) within a molecular monolayer at the liquid–solid interface without any catalyst was studied using ambient scanning tunneling microscopy. The monolayer consisted of a regular array of two species, an organic acid (trimesic acid) and an alcohol (undecan-1-ol or decan-1-ol), coadsorbed out of a solution of the acid within the alcohol at the interface of highly oriented pyrolytic graphite (HOPG) (0001) substrate. The monoester was observed promptly after reaching a threshold either related to the increased packing density of the adsorbate layer (which can be controlled by the concentration of the trimesic acid within the alcoholic solution via sonication or extended stirring) or by reaching a threshold with regards to the deposition temperature. Evidence that esterification takes place directly at the liquid–solid interface was strongly supported.

## Introduction

On-surface reactions are a widespread class of chemical reactions taking place on a surface or at an interface involving active participation of two-dimensional molecular entities. This participation is usually beyond the role of just being a solid support for the reactants.

Using scanning tunneling microscopy (STM) it is possible to actively study the elementary processes of on-surface reactions. Different types of reactions such as Ullmann coupling, imine coupling, boronic anhydridation reaction, etc. have been explored on surfaces [1–14]. In the publication by Hla et al. [1] the reaction of two single iodobenzene molecules towards one biphenyl molecule (an Ullmann reaction) on the edge of a monoatomic step of a Cu(111) substrate surface has been thoroughly investigated. In addition to the imaging, the tunnel tip was active in promoting the reaction by local energy transfer to and local transport of the reactants. Endothermal on-surface reactions of a whole molecular monolayer can be initiated by a corresponding heating process after deposition. STM imaging in different stages of the reaction has been demonstrated in such cases where the molecular entities changed their appearance due to structural and electronic changes during different reaction steps. Examples for this are the polymerization reaction of brominated copper-2,3,7,8,12,13,17,18-octabromo-5,10,15,20-tetraphenylporphyrin (CuTPPBr_8_) at an Au(111) substrate [2] or the polymerization of 1,3,6,8-tetrabromopyrene on Cu(111) and Au(111) substrates [3]. Characteristic for all these studies is that they are performed at an almost ideal monocrystalline surface in ultra-high vacuum (UHV).

On the other hand, solid–liquid interfaces are much more often encountered in real world applications ranging from heterogeneous catalysis to biomembranes. Heating is in such cases usually limited by the boiling of the liquid phase, and other means to initiate on-surface reactions are often required.

**Figure 1:**
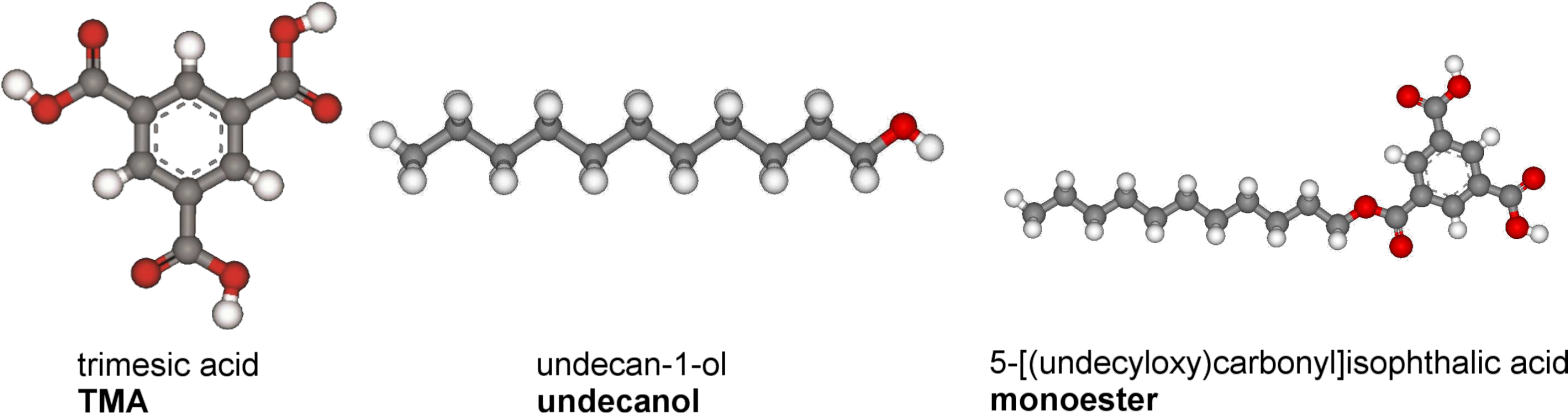
Molecular structures of the molecules involved in the study and shortened forms of their names (in bold).

Here we present a chemical reaction (esterification) between trimesic acid (benzene-1,3,5-tricarboxylic acid; TMA) dissolved in an alcoholic solvent (undecan-1-ol or decan-1-ol) on a highly oriented pyrolytic graphite (HOPG) (0001) substrate. The reaction proceeds without catalyst and is controlled by the solute concentration at the interface as well as deposition temperature. To the best of our knowledge, such a study has not yet been performed by other researchers. Ball and stick models of all the molecules used in the study are illustrated in the Figure 1.

Esterification is a chemical reaction which finds application in several areas like biology (synthesis of drug molecules), the food industry (artificial flavors and fragrances), and textiles (polyesters) [15]. The most common route of esterification starts from a carboxylic acid and an alcohol in the presence of dehydrating agents [16]. The reaction proceeds typically slow and highly reversible without a catalyst. Dehydrating agents like sulfuric or sulfonic acid [15], or milder ones like dicyclohexylcarbodiimide [17], triphenylphosphane and diazenedicarboxylate [18] are used for esterification from organic acids. In UHV, an on-surface esterification of benzene-1,4-diboronic acid and triphenylene-2,3,6,7,10,11-hexol to a 1,3,2-dioxaborole heterocycle has been studied by Zwaneveld et al. [19].

Trimesic acid (TMA) has become the “drosophila melanogaster molecule” for studies of self-assembly at crystalline surfaces both under UHV conditions [20] and at the solid–liquid interface [21–27]. Nath et al. showed the coadsorption of TMA with alcohols at an alcohol/graphite interface [26–27]. Although an ester formation is expected when mixing alcohol and acid, in situ ester formation was not found in their experiments under ambient conditions [27]. Molecular mixture at solid−liquid interfaces could possibly initiate chemical reactions and be monitored in situ with scanning tunneling microscopy (STM). Metal complexation reactions, polymerizations [28–30] and photochemical dimerization [31] are shown to be initiated at the solid–liquid interface. Initial efforts have been made to perform chemical reactions leading to covalently stabilized adlayers at metal crystal/UHV interfaces [2,10–12]. However, the size of covalently linked domains is often limited in UHV due to low diffusion of the components forming the adlayers. This problem may be easily circumvented at solid–liquid interfaces due to the high dynamics of reactants in solution. Furthermore, in this case, defects in the adsorbate layer are more often self-repaired.

## Results

**Figure 2:**
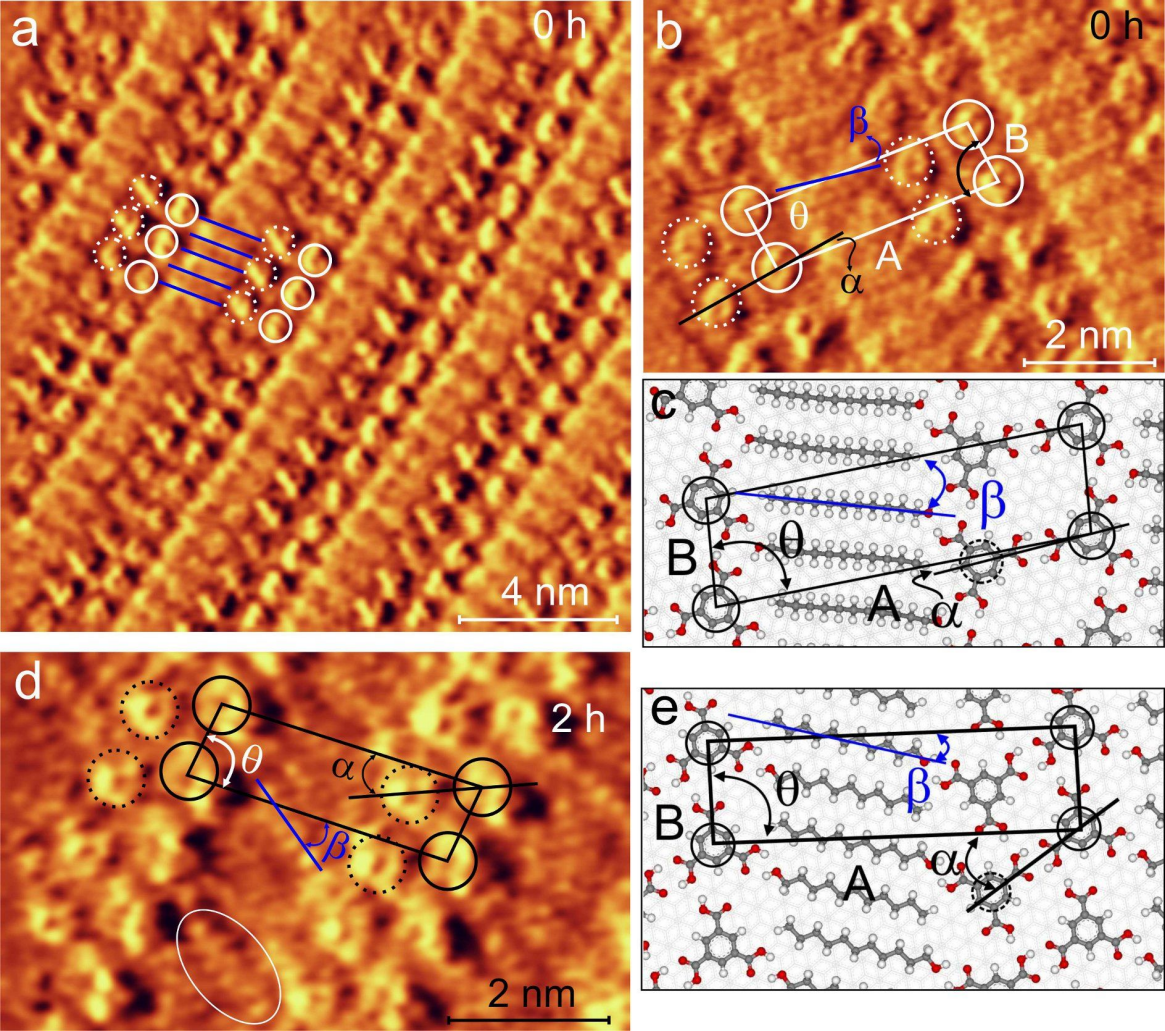
a) STM constant height image (1.2 V, 1 nA) of a TMA–undecanol linear pattern (LP0) formed on HOPG (0001) from solution without previous sonication. The LP0 consists of alternating TMA dimer tapes (dashed and full-line circle pairs) between lamellas of undecan-1-ol (blue lines); (a) is reproduced from [25], copyright 2013 Elsevier. b) A magnified section of LP0: the unit cell (parallelogram) contains two TMA and two undecanol molecules; A and B are unit cell parameters and θ is the angle embedded between them. β is the angle between the molecular axis of the undecanol back bone and the long axis of the unit cell. α describes the relative orientation of the TMA dimer with respect to the long axis of unit cell. c) Dreiding force field optimized geometric model (initial geometry based on experiments) of the LP0. d) STM constant height image (1.2 V, 1 nA) of TMA–undecanol LP2 structure formed on HOPG (0001) from solution sonicated for 2 h and the corresponding Dreiding force field optimized geometry (e). The white oval in (d) shows a submolecularly resolved undecyl chain part of undecanol.

A typical STM image of the coadsorption pattern of TMA and undecan-1-ol is shown in Figure 2a. This is consistent with the reported linear pattern (LP) of alcohol and TMA coadsorbed on the HOPG (0001) surface [26–27]. TMA interacts with undecanol via noncovalent hydrogen bonding and forms the observed LP. We call this structure LP0, where 0 indicates no previous sonication of the solution. The pattern consists of undecanol lamella (blue lines in Figure 2a [25]) and the TMA dimer tapes, which are represented by the pairs of full-line and dashed circles in Figure 2a. A magnified section of the linear pattern is shown in Figure 2b. The typical donor–acceptor double hydrogen bonds govern the interaction between TMA molecues within the TMA dimer tapes [26–27]. The unit cell parameters of this LP are A ≈ 35 Å and B ≈ 10 Å. Within the unit cell, TMA dimers form an angle of α ≈ 8° with respect to the long side of the unit cell (A). The angle θ (≈ 84°) is the angle between the unit cell vectors and β (≈ 6°) describes the relative orientation of the undecanol chain with respect to the long side A of the unit cell.

A Dreiding force field optimized structure (based on the initial geometry from experiments) of the linear pattern shown in Figure 2c is comparable with the adsorption geometry of TMA and undecanol observed in the LP. The adsorption geometry of TMA, the standard dimer hydrogen bonding motifs via carboxy groups between TMA molecules and their interaction with undecanol are discernible. The orientation of the zig-zag plane of the alkyl chain of undecanol is assumed to be perpendicular to surface according to Nath et al*.* [26–27]. The geometric parameters obtained from the simulations fit fairly well with the experiments, except for β (see Supporting Information File 1 for details). It has been shown that alkyl chains organize on HOPG in a zig-zag manner at well-defined sites [32–33]. Therefore, the difference in β observed between simulation and experiments is attributed to the interaction between molecules and the substrate, which is not included in the actual model. However, it considers the intermolecular interactions within the adlayer quite reasonably as revealed by the resemblance of the TMA dimer and undecanol lamella with the experiment.

The structures of LP formed from solutions sonicated for a longer time (at least 2 h) are noticeably different from LP0. The LP from the solution sonicated for 2 h (LP2) is shown in Figure 2d and the corresponding optimized geometry in Figure 2e. The angle between unit cell parameters A (≈34 Å) and B (≈10 Å) of LP2 remains nearly the same θ (83°) as for LP0. However, the relative orientation of the TMA dimer with respect to the long side of the unit cell in LP2 (α ≈ 23°) is clearly different from LP0 (α ≈ 8°). Additionally, undecanol molecules are tilted steeper (β ≈ 37°) compared to LP0. As a consequence, the packing density of LP2 is slightly larger than that of LP0. Undecanol molecules (one of them marked with an oval in Figure 2d) show clearly a substructure for its zig-zag plane which is parallel to the substrate.

A comparison of energetics from force field calculations (see Supporting Information File 1 for details) shows that the structure which corresponds to LP0 is energetically more favorable than LP2. This is in agreement with a previous report, where theoretical calculations showed the same result [26–27]. That is, the most favorable structure expected for TMA coadsorbed from an untreated solution in undecanol is LP0 without any external triggers. The solubility of TMA in undecanol increases upon sonication. From this solution, TMA molecules will be repelled more easily when exposed to a clean surface (HOPG) and therefore their concentration at the interface increases. UV–vis studies have confirmed such a direct correlation between sonication time and concentration (see Supporting Information File 1). Previous reports have also shown that TMA forms high packing density structures only when deposited from relatively high concentration solutions in phenyloctane and fatty acids [23–25]. That is, the energetically less favorable structure (LP2) is triggered by an external control parameter – the excess concentration at the interface.

**Figure 3:**
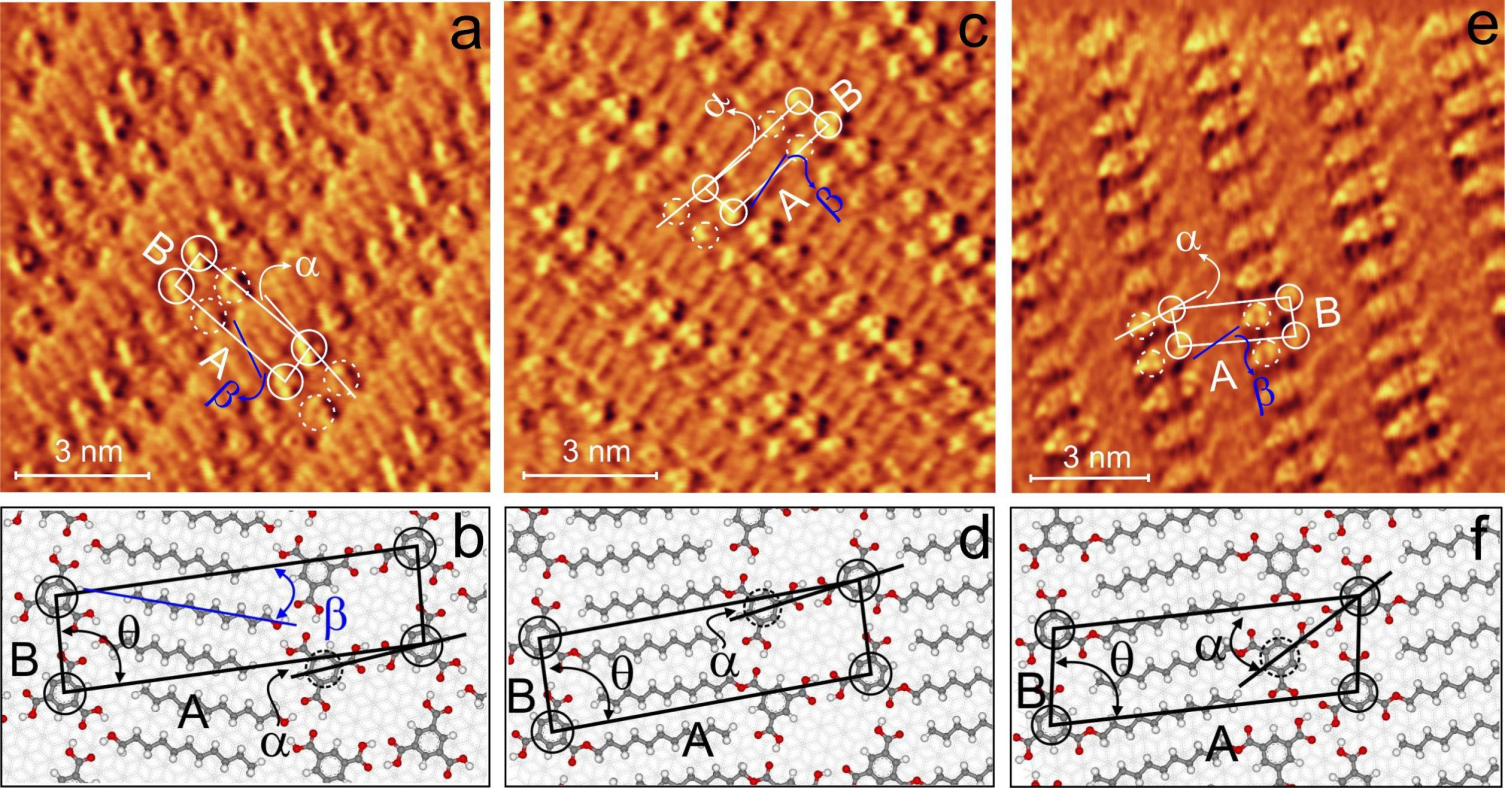
STM constant height image (1.2 V, 1 nA) of linear pattern LP4 (a), monoester type-I (c), and monoester type-II (e) deposited from a TMA–undecanol solution sonicated for 4 h. The unit cell is depicted by a parallelogram in STM images and A and B are the corresponding unit cell parameters. The blue line indicates the orientation of undecanol (a) or undecyl chains of the ester molecule (c, e) with respect to the long side of the unit cell within the lamella. Force field optimized geometries of the linear pattern (b) and patterns formed by monoesters (d, f). In the models, solid circles indicate TMA (TMA head group in the case of ester) at the corners of the unit cells and dashed circles indicate the second TMA (TMA head group) of the dimer pair.

When the sonication time is increased to 4 h, the corresponding structure LP4 (Figure 3a) quite resembles LP0 except for the orientation of the undecanol with respect to the TMA dimer (β ≈ 33°). In addition to LP4, two further close-packed structures are observed from solutions sonicated longer than 4 h (Figure 3c,e). The significant difference of these structures compared to LP4 is the shorter A-axis (a ≈12–18% reduction compared to LP0 and LP2). The geometric parameters corresponding to these structures are listed in Table 1. These additional structures cannot be interpreted in terms of coadsorption of individual TMA and undecanol molecules but of a reaction product of them which should be the corresponding monoester (this assumption will be justified later in this paper). We address these compact patterns as ester patterns in the following sections. To verify the decrease in A for the ester pattern, we have analyzed a split image (ester pattern and graphite in the same frame). The imaged graphite lattice is used as reference to scale the images and it clearly shows here that the magnitude of A is ≈31 Å (see Supporting Information File 1 for the split image).

**Table 1:** Unit cell parameters A, B, and θ orientation angles α, β, and molecular packing densities of different linear patterns (LP) and monoester patterns formed after sonication or stirring. PD is packing density (molecules/nm^2^).

Sonication time (h)	0	2	4		Synthesized monoester
Stirring time (h)	0	10	15		
	LP0	LP2	LP4	Ester4^d^	Ester pattern

A (Å)^a^	35	34	36	31 (28)	29
B (Å)^a^	10	10	10	10 (10)	10
θ (°)^b^	84	83	86	83 (83)	83
α (°)^b^	8	23	8	4 (25)	25
β (°)^b^	6	37	33	7 (27)	28
PD^c^	0.58	0.61	0.54	0.65 (0.71)	0.65

^a^Distances may have an error of ±2 Å, ^b^angles may have an error of ±2°, ^c^PDs may have an error of ±0.2 nm^−2^, ^d^the numbers in parenthesis correspond to values for type-II monoester (see Figure 2e).

There are two ester patterns visible on the surface which are slightly different in their value of A and significantly differ for the relative orientation of the head groups of the monoester (α; dimer formed by the TMA group of ester) and undecyl lamella (β; with respect to the long side of unit cell). We refer to the ester at the interface with A ≈ 31 Å as ester4-type-I and with A ≈ 28 Å as ester4-type-II in the following sections. The Dreiding force field optimized geometries of these ester structures (based on the initial geometry from the experiment) are shown in Figure 3d,f. The adsorption geometries of type-I and type-II esters (except β) are comparable with the experiments. The relative orientation of the head group of TMA and undecyl chain of the ester patterns resembles LP0 and LP2. This is very likely connected with the original linear patterns from which each type of these ester patterns has developed. Ester4-type-I has a lower packing density than ester4-type-II, which also holds for LP0 and LP2. Further geometrical details of the simulation are provided in Supporting Information File 1.

The optimized structure corresponding to LP0 (Figure 2c) shows that within the linear pattern even closer packing is not possible. This is hindered by steric repulsion between the methyl end groups of undecanol and the hydrogen atoms of the C–H groups of TMA. A closer packing is only possible via reorganization of TMA dimers as seen in LP2 or via a gauche isomer of undecanol parallel to the surface. However, the undecanol molecules are observed as linear features in the STM images, which is consistent with their linear zig-zag geometry. That is, the observed decrease in A could be explained only by the replacement of TMA and undecanol by the corresponding monoester at the interface. The theoretically calculated distance between neighboring molecules in ester patterns is 19–24% shorter than that in various LPs. This is consistent to the observed reduction of ≈12–18% in A for the ester pattern compared to LPs. That is, ester formation only can further increase the packing density. The geometric pattern of the ester molecules here resembles the adsorption structure of hexadecyl ester on a Au(111) surface [34].

We then further extended the sonication time up to 8 h. Both ester pattern type-I and type-II are obtained from solutions sonicated for 6 and 8 hours as well. The structure of these ester patterns remains nearly unchanged when the sonication time is increased. However, the A value of LP increases slightly as the time of sonication increases, whereas the other geometrical parameters (e.g., B, θ) remain nearly unchanged. As a result, the packing density of LP decreases slightly with increasing sonication time. It is to be noted that the distances between TMA dimers in the dimer tape of LP and the dimer of TMA head groups in ester pattern type-I and type*-*II remain the same for all sonication times. That is, the dimers are always intact and with sonication only their relative orientation with respect to the TMA tape changes in different structures (Table 1).

**Figure 4:**
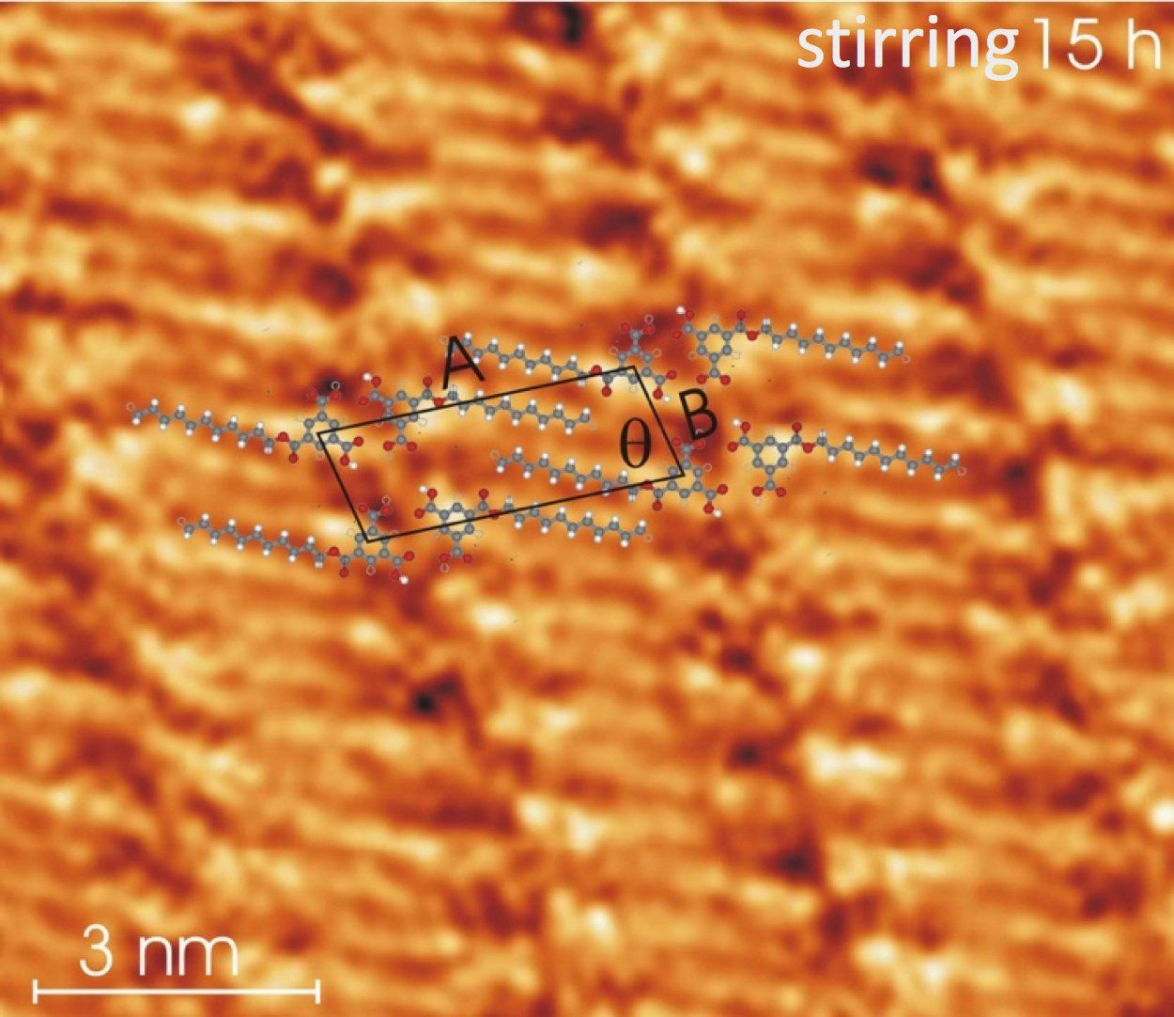
STM constant current image (1.1 V, 1 nA) of TMA–monoundecyl ester type-I obtained from a TMA–undecanol solution stirred for 15 h**.** The corresponding unit cell parameters of the above pattern are A = 29 ± 1 Å, B = 10 ± 1 Å.

Practically the same ester patterns are observed also for two alternative preparation methods: prolonged magnetic stirring for about 15 h or increasing the substrate temperature to 60–80 °C during deposition. As an example, the STM image of TMA–monoundecyl ester type-I obtained from the TMA–undecanol solution stirred for 15 h is shown in Figure 4.

**Figure 5:**
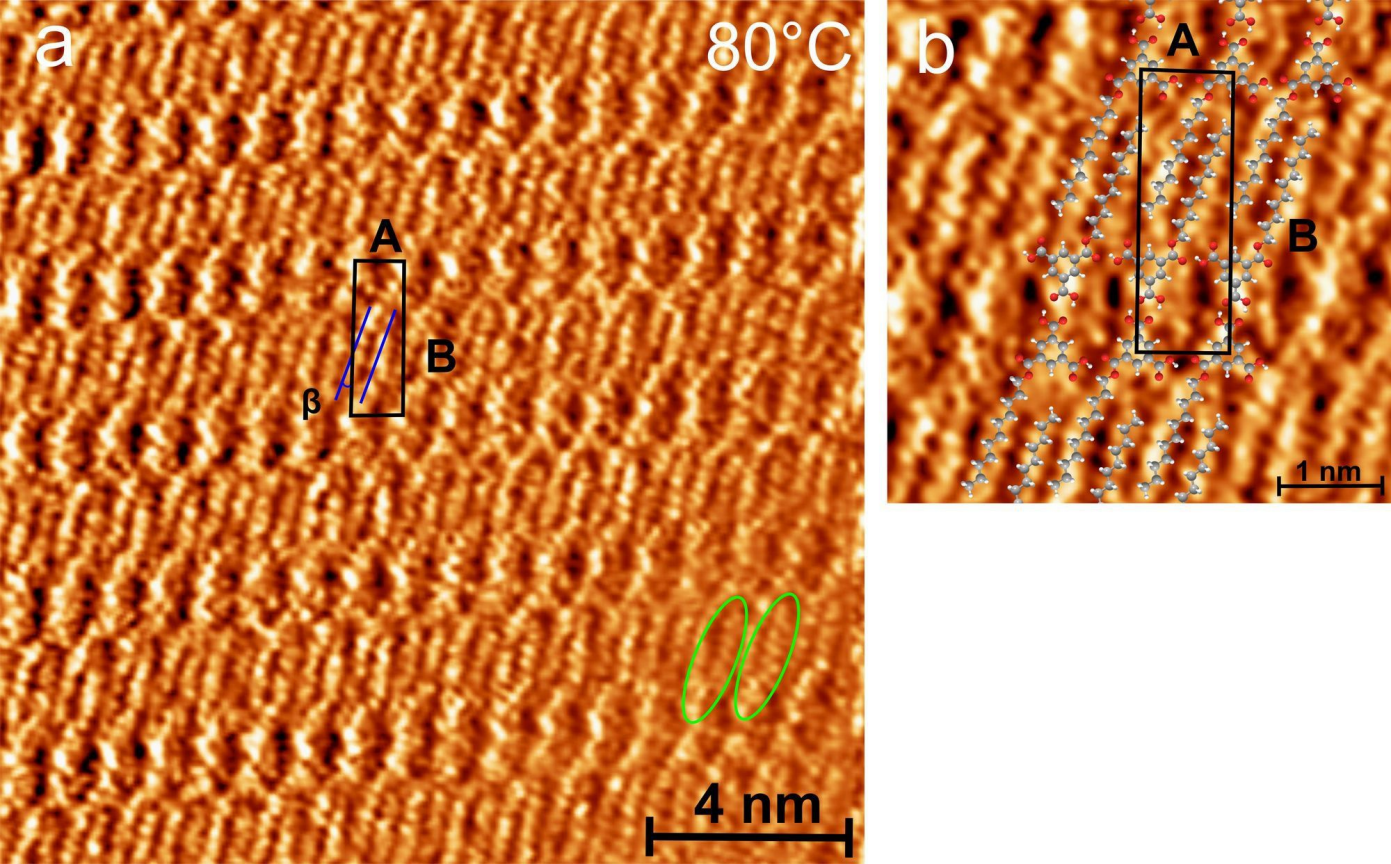
STM constant height images (1.2 V, 1.3 nA) of monoester obtained from a TMA–undecanol solution on HOPG (0001) deposited at ≈60–80 °C. (a) The parallelogram depicts the unit cell of the ester pattern where A and B are the corresponding unit cell parameters. (b) The force-field-optimized geometry of the monoester pattern overlaid on the STM image (5 × 5 nm^2^).

The ester pattern observed after increasing the substrate temperature during deposition (subsequent STM was carried out at room temperature) is shown in Figure 5. The unit cell parameter A = 3.0 ± 0.1 nm and the angle of the undecanol alkyl chain with respect to A, β = 16 ± 3°, are obtained from the STM images. With respect to most of the structural parameters, the patterns correspond (see Table 1) to that observed for sonication and stirring. This type of preparation procedure has been previously reported and leads also to an increased concentration in the deposited solution due to enforced evaporation as well as to an increased mobility of the molecules [35].

**Figure 6:**
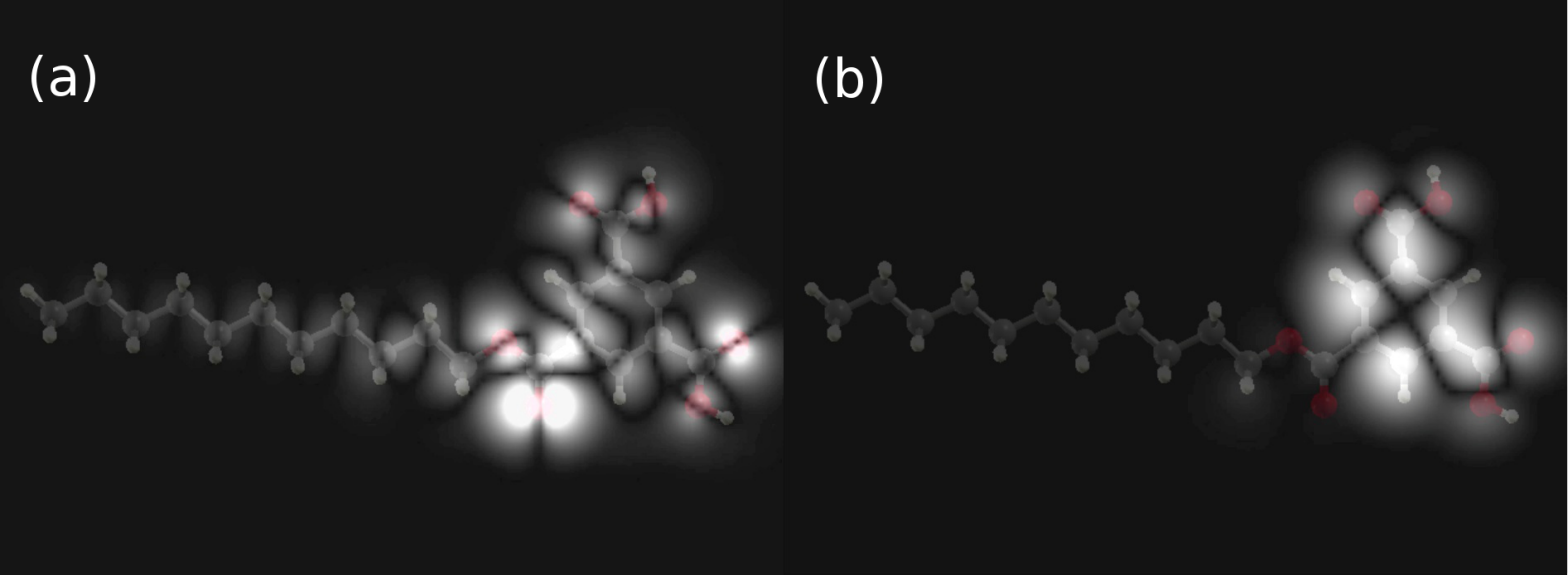
Simulation of the STM constant height mode images (HOMO (a) and LUMO (b)) of a single monoester molecule.

Figure 6 shows simulated constant height mode STM images obtained from a calculated local density of states (LDOS) of the free monoester molecule. Both HOMO and LUMO show a strong intensity close to the location of TMA and binding to the alkane chain. Such a characteristic feature can be observed also in the experimental STM images of Figure 4 and Figure 5. Of course, most of the details from the calculation cannot be expected to be well reproduced in an experimental STM images due to the approximation of isolated molecules neglecting the adsorbate–substrate interaction as well as various effects of the tip and the environment on the imaging.

**Figure 7:**
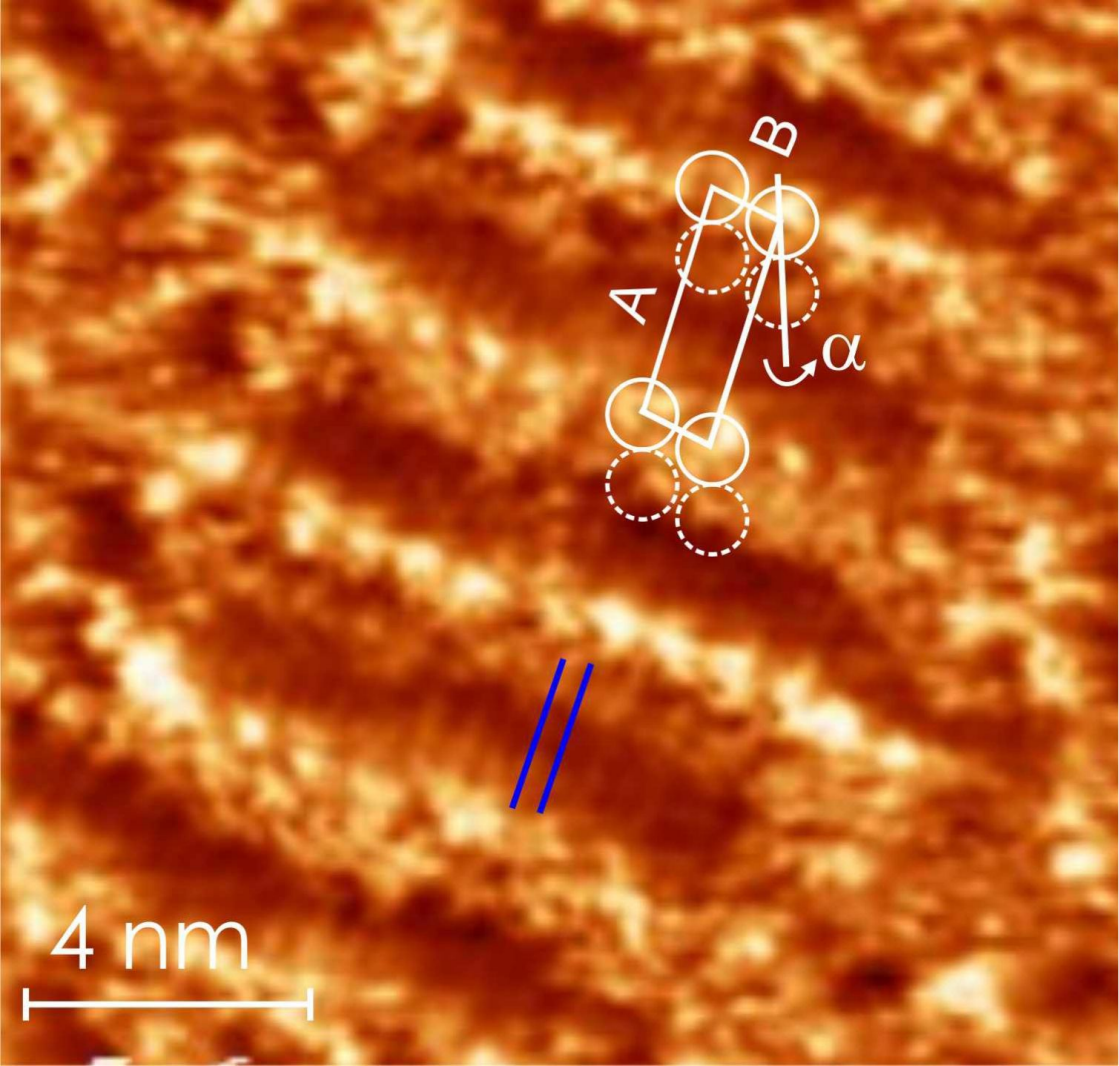
STM constant height image (1.2 V, 1 nA) of the linear pattern of the synthesized monoester at the HOPG–undecanol interface (A = 3.1 ± 0.1 nm; B = 1.0 ± 0.1 nm; α = 23 ± 1°). The triangular features indicated with circles correspond to the TMA head groups and blue lines indicate alkyl chains of synthesized monoester molecules. The orientation of the alkyl chain adopts an angle of ≈2° with respect to the A axis (indicated as β in other images). According to the calculation it is expected that the alkyl chain of synthesized monoester should be almost parallel to the A axis.

To further prove that the patterns observed at high sonication/stirring time and high substrate temperature really show deposited monoester molecules, we have studied the self-assembly of the synthesized monoester (see Supporting Information File 1) dissolved in undecanol as a reference experiment. The monoester was synthesized according to literature [36]. Figure 7 shows a self-assembled pattern of the synthesized monoester deposited at the HOPG–undecanol interface (the concentration of the solution should be considerable less than 8 × 10^−3^ M (there were sediments of molecules at the bottom of the vial). The circles depict the location of TMA groups of the synthesized monoester. A and B are the unit cell parameters and θ is the angle between them. α depicts the angle between the molecular unit cell axis A and the pair formed by TMA groups of adjacent monoesters. These quantities are indicated in Table 1. The geometrical parameters of the synthesized monoester pattern obtained here are in excellent agreement with those observed for ester type-II formed from the TMA–undecanol solutions at high sonication or stirring time or enhanced substrate temperature. This experiment establishes that the observed close packed patterns (ester type-I and -II) obtained at the TMA–undecanol interface from solutions at high sonication/stirring time and high substrate temperature are made of monoester molecules.

To better understand the general behavior of ester formation in aliphatic alcohols, we have investigated the ester formation using decan-1-ol as an alternative solvent. Details of the LPs formed at different sonication times in this solution are provided in Supporting Information File 1. As in the case of undecanol, at low sonication times, for LPs of coadsorbed TMA and the alcohol molecules are formed. Different types of monoesters (type-I and type-II) are observed from solutions that were sonicated for four hours. After esterification, the packing density of LPs decreases as the sonication time increases. These results are all very similar to those obtained for the undecanol–TMA mixture. This shows that the concentration driven LPs and ester formation are very likely common for TMA and long chain alcohols such as decanol and undecanol.

## Discussion

**Scheme 1:**
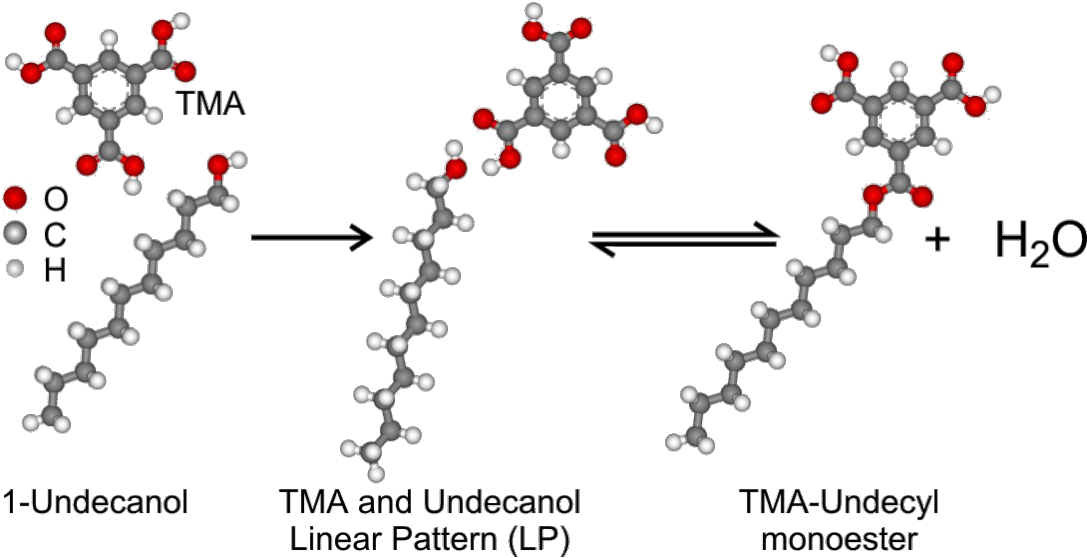
Proposed scheme of ester formation from TMA and undecanol via an intermediate dimer.

Generally, esterification is a reversible process (Scheme 1) and the yield is low without dehydrating agents [15–18]. To increase the yield, Le Chatelier's principle is commonly used; that is, the concentration of one of the reactants is increased. This is supported by the molecular collision theory. The higher the molecular concentration, the more collisions of suitable pairs of molecules can take place. The successful collisions should have also sufficient activation energy transferred at the moment of impact to break the existing bonds and to form new ones, resulting in the reaction products.

**Figure 8:**
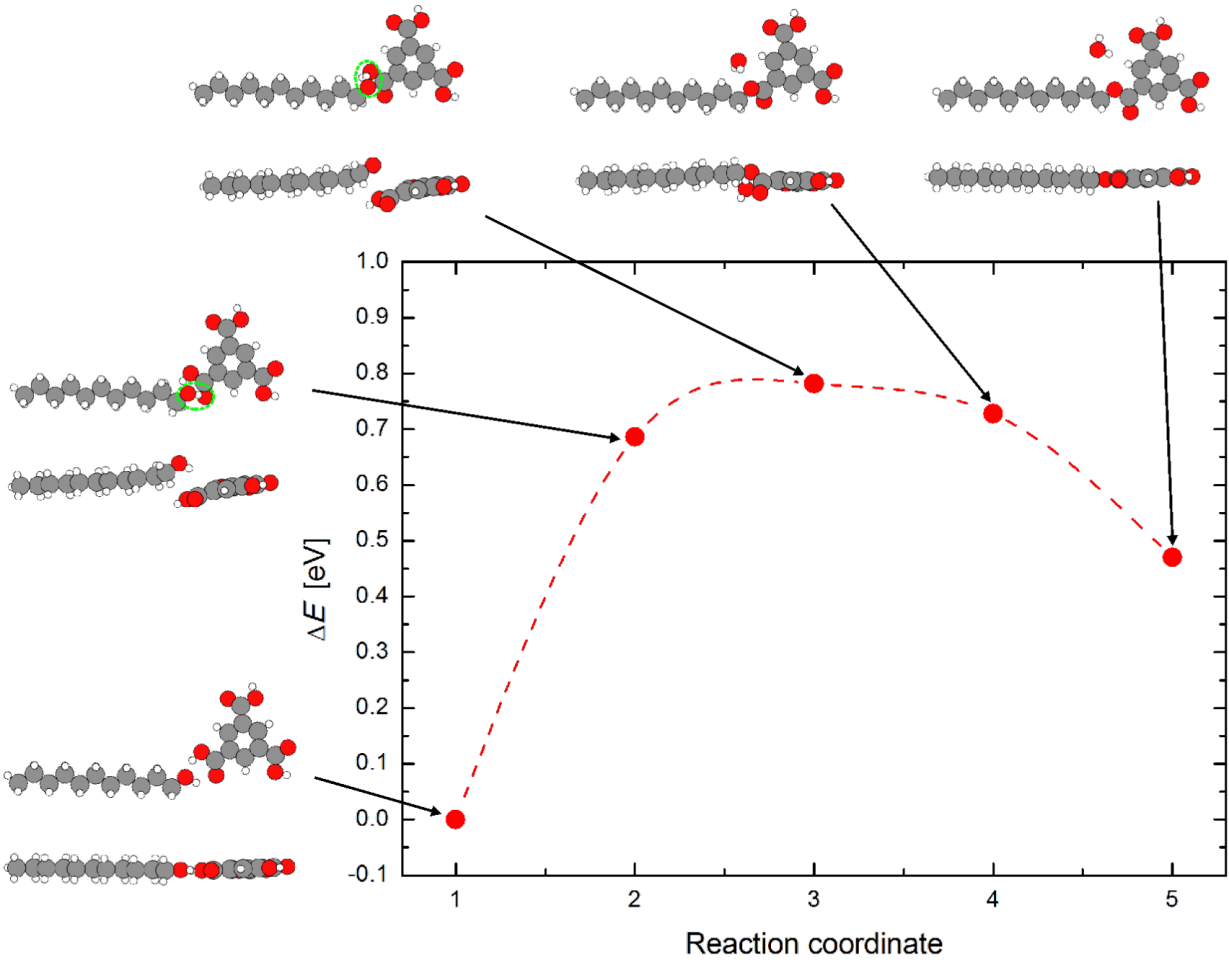
Energy diagram of the reaction path of TMA and undecanol to form TMA–undecyl ester and water for isolated molecules (gas phase) calculated using DFT (PBE). The corresponding geometry of the molecules in the simulation is shown for each energy point marked in the reaction path. The “flat approach” (almost 2-dimensional) as shown in the simulation for the free molecules is strongly supported by the underlying crystal surface in the experiments.

Scheme 1 shows a proposed reversible esterification route with a dimer intermediate of TMA and undecanol to TMA–monoundecyl ester. The possible formation of TMA–monoundecyl ester and water from a TMA–undecanol dimer in the gas phase (see energy diagram in Figure 8) was simulated. To simulate the reaction path and energy barrier, a "nudged elastic band" calculation was done using DFT with the program code GPAW [37]. The molecule was placed in a large box with non-periodic boundary conditions and 7 Å of vacuum in each direction. The starting and end geometry (reactants and products) are first optimized separately, then three intermediate geometries are interpolated and the whole path of five reaction steps is relaxed together to find the lowest energy barrier. The XC-functional PBE [38], a LCAO dzp basis set, and default values for the self-consistency cut off were used. The starting and end geometry (reactants and products) are first optimized separately, then three points are interpolated and the whole path is relaxed together to find the lowest energy barrier.

The formation energy calculated for the monoester from undecanol and TMA is, *E*_ester_ − (*E*_TMA_ + *E*_undecanol_ − *E*_water_) ≈ 470 meV with a reaction barrier of ≈800 meV. It is interesting to note that the reaction mechanism shows several intermediate steps that involve a nearly planar geometry of TMA and undecanol before the monoester formation. Therefore, we suggest that the coadsorption pattern (LP) allows the reactants to approach each other already in a quite favorable relative orientation for a subsequent reaction. That is the “flat approach” (almost 2-dimensional) necessary as shown in the simulation as the free molecules correspond fairly well also to the experimental situation of the on-surface reaction considered experimetally. The equilibrium that is established between ester and TMA–undecanol dimers at the interface can be influenced if excess reactants are offered at the interface. That is, monoester formation can be controlled by varying the concentration of the acid. By increasing the sonication time and stirring, the concentration of TMA is increased in the solution. Furthermore, when this solution is applied on a fresh HOPG surface, the additional TMA molecules within the solution tend to become preferentially accumulated at the interface. This increase in the concentration of TMA and the interface, together with its flat adsorption geometry (templated by the planar substrate surface), favors the esterification reaction to proceed forward.

Typically, the formation of an ester is promoted at high temperature. The effect of changing the temperature on the equilibrium, and thereby changing the heat in the system, can be understood by including heat energy in the reaction formula either on the side of reactants or the products. According to Le Chatelier's principle, an increased temperature would then favor the forward reaction of esterification similar to increasing the relative concentration of the reactant. Enhanced temperature can also result in an increased evaporation of water that is created in this reaction, thereby removing it and pulling the equilibrium to the side of the ester. On the other hand, aqueous systems help the equilibrium to be established in the reverse direction by providing an excess of the water needed for the hydrolysis. The temperature influence on a chemical reaction at the liquid–solid interface by STM is also reported by Hipps et al., that is, the formation of an ester promoted by high temperatures [39].

There remain three possible hypotheses concerning the origin of the ester or the location of esterification, respectively: 1) The ester molecules originate all from the solution (either as contamination or as the result of an esterification in the bulk liquid phase); 2) the seed molecules for the ordered adsorbed ester pattern originate from the solution but around them further esterification takes place at the interface; or 3) the esterification observed here is a typical on-surface reaction.

Concerning 1): ESIMS analysis (see Supporting Information File 1) showed some traces of the ester after sufficiently long sonication time due to an initiated reaction in the bulk of the solution. Furthermore, taking into account the detection limit of the method, there should be sufficient preexisting ester molecules in the droplet to enable a complete coverage of the substrate by an ester monolayer (ester pattern). Nevertheless, this hypothesis can be ruled out, since there is a threshold (for sonication/stirring time as well as deposition temperature) to find the monoester pattern. The threshold indicates a critical concentration of TMA in the solution within three different experimental approaches: sonication, stirring, and deposition at enhanced temperature. If the ester formed in the solution would be the origin of the ester pattern, then this pattern should be observed at lower sonication or stirring time or deposition temperature as well. We also note that sonicated solutions retained for several days (12 days) did not show any ester pattern (see Supporting Information File 1 for details of this experiment). This indicates a finite lifetime for the higher concentrated (possibly super-saturated) solution after which we observe only a linear pattern of TMA and undecanol (and no ester pattern). This would not be the case if the ester pattern originates from ester formed due to sonication within the solution.

Furthermore, the reference experiment with a solution of the monoester (which definitely had a much higher concentration of monoester molecules than the solutions discussed for the case 1) did verify the corresponding adsorption pattern but did not lead to a comparatively large ordered area of the pattern as had been found in the previous experiments (TMA–undecanol with 4 h sonication). Evidently, only some growth directly on the substrate could explain the experimental findings, and hypothesis 1) can be ruled out completely.

Concerning 2): There would definitely always be sufficient monoester molecules available in the solution – especially after the corresponding treatments (sonication, stirring or heating, respectively) to enable single-molecule adsorption with a subsequent growth process around a seed molecule coming from the solution. However, once again, such a process cannot explain the threshold behavior found in the deposition experiments here.

**Figure 9:**
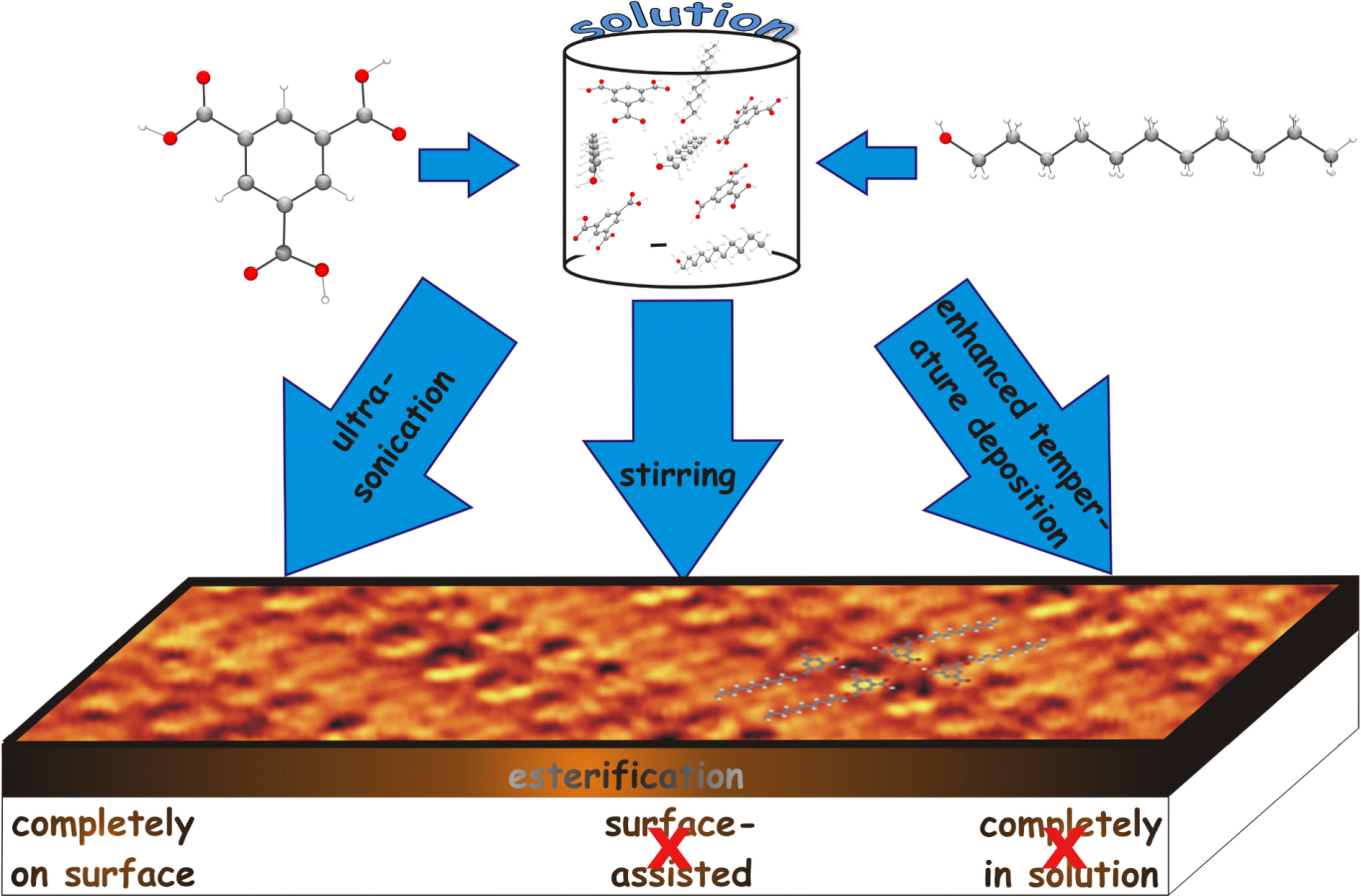
Three possible hypotheses for the formation of the monoester from TMA and undecanol.

Concerning 3): An on-surface reaction can explain the concentration thresholds found which shifts the reaction balance towards the production of the ester by effectively increasing the concentration on the surface. This leads to an increased packing density of the coadsorbed reaction partners. Furthermore, their mutual arrangement, especially in the LP2 pattern, creates a good precondition for the final reaction initiated by something as a two-dimensional pressure. This drives the adjacent reaction partners even closer to each other with increasing packing density. Furthermore, the simulated reaction path of TMA–monoundecyl ester (Figure 8) does not only illustrate this statement, but also nicely shows how the role of the supporting planar substrate dramatically reduces the amount of mutual spatial configurations of the reaction partners in a very favorable way. We note that no other assembly (particularly any disordered phase) than the well-ordered LP and ester patterns are observed at the interface of all solutions. As observed using ESIMS, the sonicated solutions possess minor amounts of monoester, diester and decarboxylation products. If these products would be the origin of the assembly at the interface, one should expect only a disordered phase, which is not the case in the experiments. Possible sonochemistry products formed in the solutions are most likely stabilized within the solutions and do not appear at the interface. After these discussions, hypotheses 1) and 2) can be ruled out and 3) is assumed as summarized in Figure 9.

As an outlook, we suggest to use appropriate diols to create covalently bound 2-dimentional networks made of the corresponding polyesters. For the present system based on TMA, this could lead to 3- and/or 6-fold symmetric networks in contrast to 4-fold symmetric networks which have been already fabricated based on porphyrines.

## Conclusion

In summary we have investigated the molecular self-assembly from a solution of TMA in undecanol at the HOPG–undecanol interface. Above a critical concentration of TMA, which can be controlled by the time of sonication/stirring of the mixture of TMA and undecanol, a monoester is formed at the interface. A similar result was also observed by increasing the deposition temperature. To prove this assumption, we have also investigated the self-assembly of presynthesized monoester molecules at the undecanol–HOPG interface and observed a very similar pattern as obtained before. The monoester formation has been interpreted as an on-surface reaction. We believe that this result will initiate further work towards covalently bound ultra-thin surface coatings.

## Experimental

TMA (0.05 g) and undecanol (7.5 mL, Aldrich, 98%) were mixed leading to a clear solution and a sediment of excess TMA at the bottom. Next, these samples were sonicated (1–8 h) or stirred (1–30 h). Afterward, the mixtures were either centrifuged or allowed to rest undisturbed for one day. From the optically clear supernatant 2 μL were put on a freshly cleaved HOPG (0001) basal plane substrate and in situ investigated using STM mechanically cut Pt(80)/Ir(20) tips. During imaging, the tip apex is introduced into the droplet deposited at the HOPG substrate. The figure captions of the STM images contain the imaging parameters for tunnel bias and current, respectively.

The HOPG (0001) substrate was preheated up to 60–80 °C, then a droplet of 2 µL of unprocessed TMA–undecanol solution was applied on this preheated substrate. The sample was kept at that temperature for 10 min and then the substrate was cooled down to room temperature for STM imaging.

DFT calculations were carried out using the grid-based projector augmented wave method (GPAW) [37]. The PBE exchange-correlation functional [38] and the LCAO mode [37] with the standard double-zeta-polarized (dzp) basis set of atomic orbitals was used. The reaction path was modeled by a “nudged elastic band” (NEB), whereby each step was fully relaxed.

## Supporting Information

File 1 Additional experimental results.
Dreiding force field calculations of different types of linear patterns of TMA and monoester on graphite double layer, UV–vis spectra as a function of sonication and concentration for different sonication times, a split image of the ester pattern and graphite, the synthesized monoester assembly pattern at the HOPG–undecanol interface, NMR and ESIMS spectra of the synthesized TMA–monoundecyl ester, evidence of self-assembly out of a solution of TMA in decanol controlled by concentration, ESIMS data of the ultrasonicated solution of TMA and undecanol-1, and the time-dependent evolution of LP and ester pattern.
