# Supplementary material for: Ester formation at the liquid–solid interface
Source: Beilstein J Nanotechnol. 2017 Oct 12;8:2139–50. doi: 10.3762/bjnano.8.213 (PMC5647715; doi:10.3762/bjnano.8.213)
Supplement: File 1 — Additional experimental results. Dreiding force field calculations of different types of linear patterns of TMA and monoester on graphite double layer, UV–vis spectra as a function of sonication and concentration for different sonication times, a split image of the ester pattern and graphite, the synthesized monoester assembly pattern at the HOPG–undecanol interface, NMR and ESIMS spectra of the synthesized TMA–monoundecyl ester, evidence of self-assembly out of a solution of TMA in decanol controlled by concentration, ESIMS data of the ultrasonicated solution of TMA and undecanol-1, and the time-dependent evolution of LP and ester pattern. [file Beilstein_J_Nanotechnol-08-2139-s001.pdf]

Supporting Information

for

Ester formation at the liquid–solid interface

Nguyen T. N. Ha*[1], Thiruvancheril G. Gopakumar*[2], Nguyen D. C. Yen[1], Carola Mende[3], Lars Smykalla[1], Maik Schlesinger[4], Roy Buschbeck[3], Tobias Rüffer[3], Heinrich Lang[3], Michael Mehring[4], and Michael Hietschold[1]

Address: [1]Solid Surfaces Analysis Group, Institute of Physics, Technische Universität Chemnitz, D-09107 Chemnitz, Germany, [2]Department of Chemistry, Indian Institute of Technology Kanpur, Kanpur 208016, India, [3]Inorganic Chemistry, Institute of Chemistry, Technische Universität Chemnitz, D-09107 Chemnitz, Germany, and [4]Coordination Chemistry, Institute of Chemistry, Technische Universität Chemnitz, D-09107 Chemnitz, Germany

* Corresponding author

Email: Nguyen T. N. Ha - thi-ngoc-ha.nguyen@physik.tu-chemnitz.de; Thiruvancheril G. Gopakumar - gopan@iitk.ac.in

Additional experimental results

Table S1: Dreiding force field calculations of different types of linear patterns of TMA and mono ester on graphite double layer.

Structure	Model	Geometrical parameters	Energy gain per TMA molecule in LPs compared to a free TMA molecule on graphite (eV/molecule)
Linear pattern comparable to no sonication (LP0).	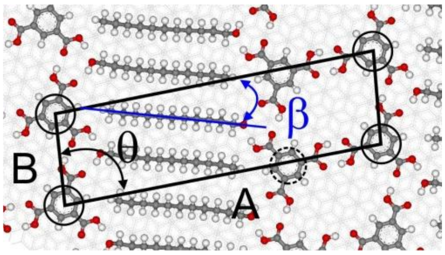	A = 33.78 Å<br>B = 9.63 Å<br>θ = 84.5°<br>α = 3°<br>β = 17.2°<br>*PD = 0.614 molecules/nm^2	-1.12
Linear pattern comparableto no sonication (LP0_1). Relative rotation of undecanol with respect to graphite plane is different in this case.	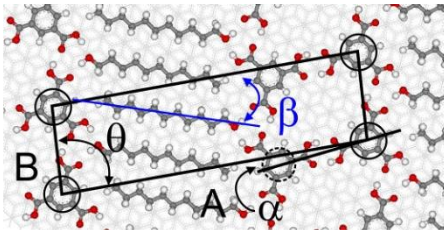	A= 33.49Å<br>B =9.48Å<br>θ=85.8°<br>α = 6.3°<br>β = 18.3<br>*P.D = 0.629 molecules/nm^2	-1.12
Linear pattern comparable to 2 hours sonication (LP2)	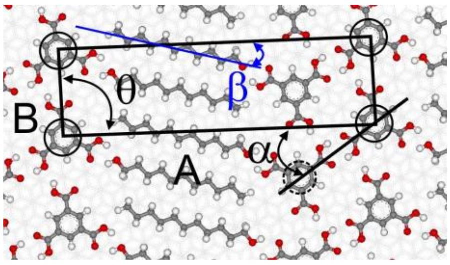	A= 33.49 Å<br>B= 9.31 Å<br>θ= 88.5°<br>α = 33.7°<br>β = 15.6<br>*P.D = 0.641 molecules/nm^2	-0.97

Linear pattern comparable to 4 hours sonication (LP4). This structure is slightly loose packed compared to LP0_1.	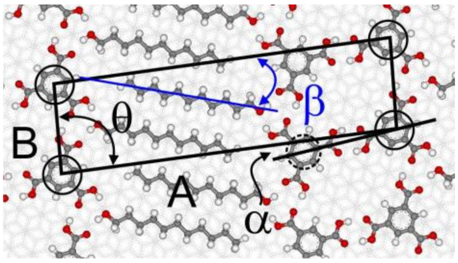	A = 35.44 Å<br>B = 9.53 Å<br>θ= 86.4°<br>α = 6.3°<br>β= 17.3<br>*P.D = 0.592 molecules/nm^2	-1.10
EstertypeI	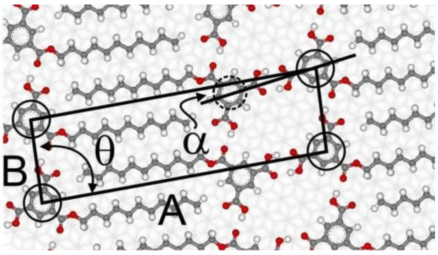	A = 32.35 Å<br>B = 9.46 Å<br>θ= 87°<br>α = 6.9°<br>*P.D = 0.653 molecules/nm^2	-2.09
EstertypeII	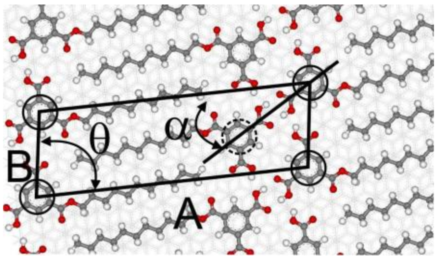	A = 29.91 Å<br>B = 9.33 Å<br>θ= 82.2 °<br>α = 30.5°<br>*P.D = 0.716 molecules/nm^2	-1.90

*P.D. is packing density (molecules/nm^2).

The geometry of each structure is optimized using Drieding force field implemented in DS ViewerPro. The starting point of each structure is based on experimental geometry parameters. Summary of all calculations is provided in Table S1. The absolute energy of different structures is dependent on the calculation methods used [1,2]; however, the relative energy of different type of LP/Ester within a single method is useful for comparison. A, B are the unit cell parameters, θ is the angle embedded between A and B of optimized structures. The output parameters of different optimized models were compared to the corresponding experimental parameters to finalize the models.

The model with a maximum of parameter matching to an experimentally observed structure is assigned to it. Optimized LP corresponding to no sonication is similar to LP0 and LP0_1 and that at higher sonication time is LP2. LP0 is energetically ≈ 150 meV more stable than LP2. That is LP0 is energetically more favorable thanLP2, which is observed spontaneously (reported before for TMA-undecanol mixture [3,4]). LP2 is formed only by triggering with external stimuli namely high concentration of TMA at the interface. LP4 is a relatively stable pattern compared to LP2, however, it is slightly looser packed. Ester typeI is comparable with LP0 and typeII is related to LP2. The relative energies and packing densities of these ester types are comparable with the corresponding LP.

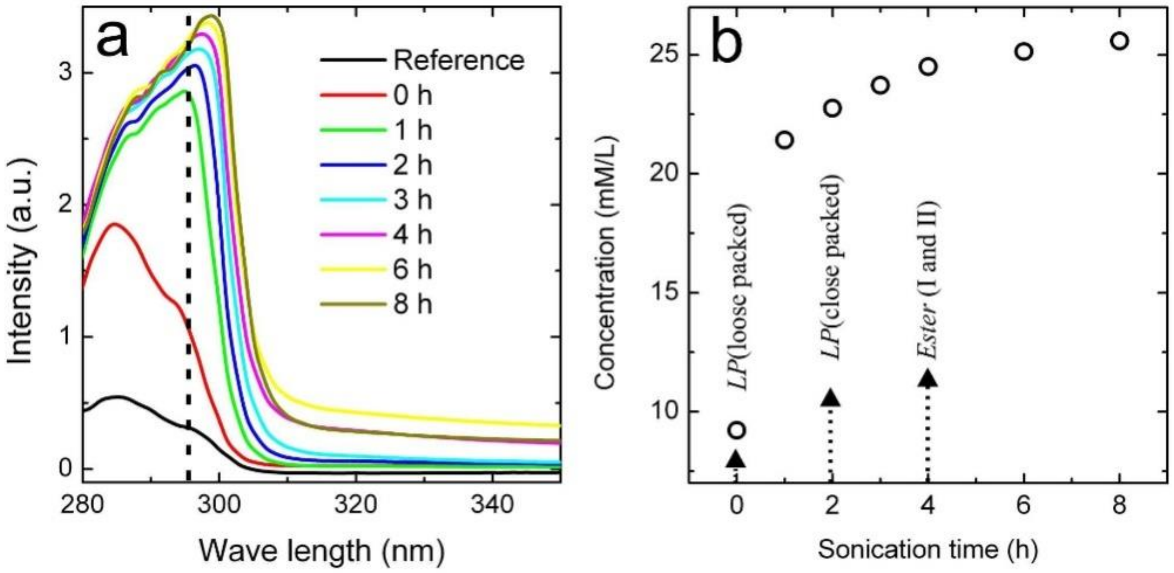

Figure S1: a) UV-vis spectra of TMA in undecanol at different sonication times. Reference is a solution with known concentration. 0 hours (0h) indicate solution which has been not sonicated. Dashed line indicates the main peak at ≈ 296 nm, which is used for estimating the concentration of solutions sonicated for different times. Concentration of solution shows a quick increase upon starting sonication, further increase is slower and eventually saturates. A relatively weak shift of ≈ 5 nm is

observed for the main peak (294–299 nm) with increasing sonication time. b) Concentration as a function of sonication time.

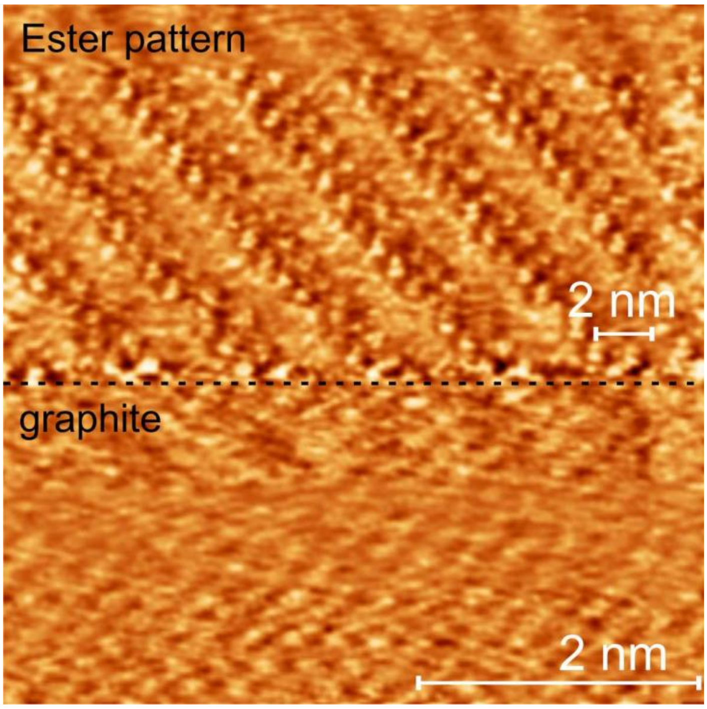

Figure S2: STM constant height image (1.2 V and 1.0 nA) of graphite and *Ester* pattern in same frame (split image). The dashed line separates graphite and ester pattern and is obtained by changing a resolution factor at the indicated line. Scale of the top part of the image is 5 times that of the lower part.

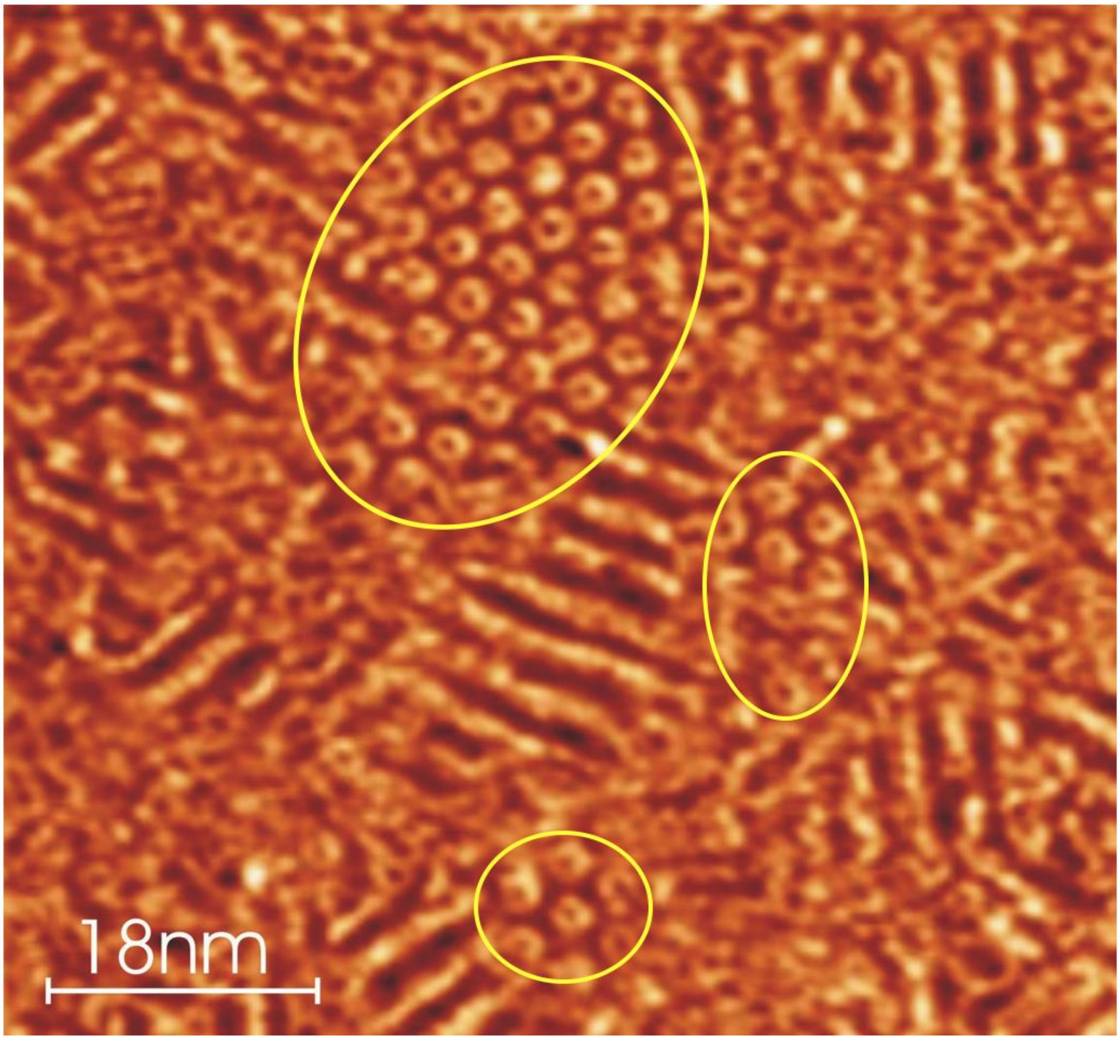

Figure S3: STM constant height image (1.2 V, 1 nA) of synthesized monoester linear pattern at HOPG-undecanol interface in the large scan area. Self-assembled structures of monoester are observed as islands in this scan area. The STM image clearly shows an adsorption structure of the monoester as found developing from the coadsorption pattern of TMA and undecanol as well as a further hexagonal structure (yellow ovals), which is not the object discussed in this paper.

In analogy to reference [5], benzene-1,3,5-tricarbonyl trichloride was treated in CH_2Cl_2 solution with undecanol in the presence of pyridine as acid scavenger. After aqueous work-up and subsequent column chromatography the TMA-monoundecyl ester could

be obtained in analytically pure form. For further infomation cf. reference [6]. Below the ^{1}H NMR and ESIMS spectra of the TMA-monoundecyl ester are given.

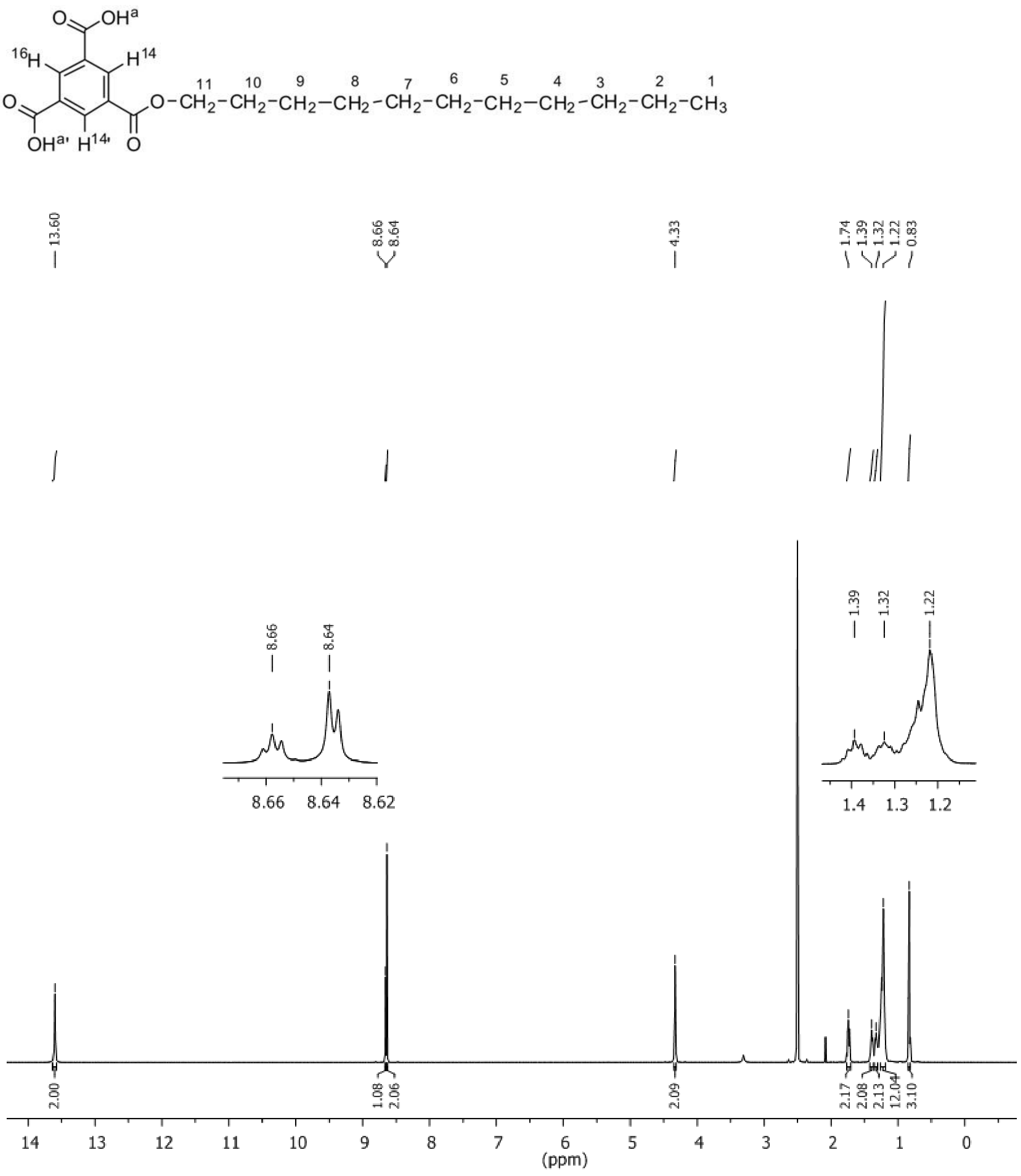

Figure S4: ^{1}H NMR and ESIMS spectra of the TMA-monoundecyl ester.

^{1}H NMR [$(CD_3)_2SO$), ppm]: δ = 0.83 (m, 3H, H^1), 1.22 (m, 12H, $H^{3,4,5,6,7,8}$),1.32 (m, 2H, H^2), 1.39 (m, 2H, H^9), 1.74 (m, 2H, H^{10}), 4.33 (m, 2H,H^{11}), 8.64 (m, 2H, $H^{14,14'}$), 8.66 (m, 1H,H^{16}), 13.60 (s, 2H, $H^{a,a'}$).

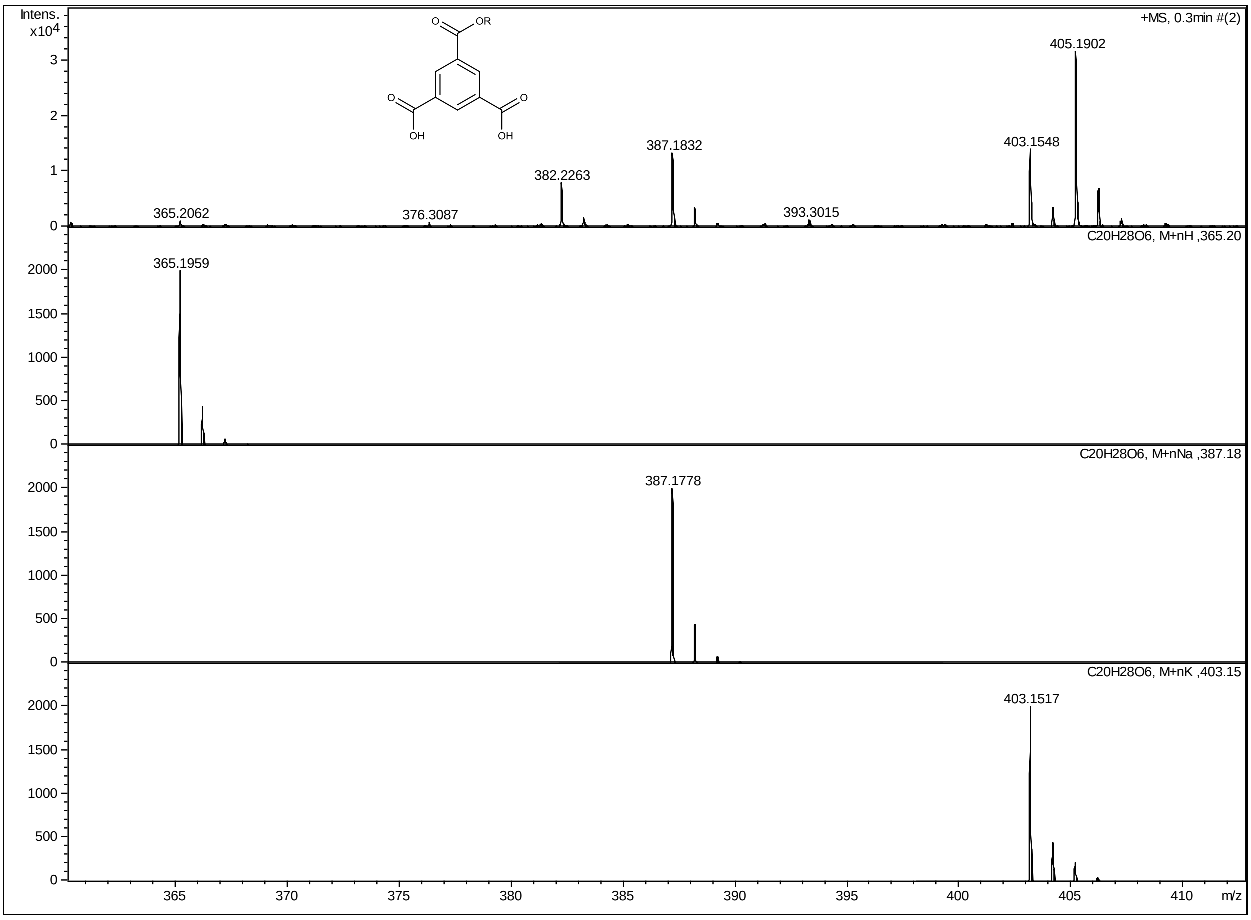

Figure S5: Part of ESIMS spectrum of the TMA-monoundecyl ester (5-[(undecyloxy)carbonyl]benzene-1,3-dicarboxylic acid, R = $C_{11}H_{23}$) showing the region of the proton, sodium- and potassium-ion adducts (measured (above), calculated (below)). Spectrum was measured using acetonitrile as solvent.

Table S2: Summary of geometric parameters, A, B, θ, ß and molecular packing density of different linear patterns and Ester patterns.

Sonication time (h)	0	4		6		8	
	L.P.	L.P.	Ester#	L.P.	Ester#	L.P.	Ester#
A(Å)*	32	33	28(25)	35	28(25)	37	28 (24)
B(Å)*	10	10	10(10)	10	10(10)	10	10(10)
θ(°)**	85	87	85(82)	89	89(78)	86	86 (80)
β(°)**	17	14	22	15	24	16	26 (15)
P.D.	0.63	0.61	0.72	0.57	0.71(0.82)	0.54	0.74(0.84)

* Distances have an error margin of ±1 Å, **Angles have an error margin of ±2°, # parenthesis shows the corresponding values for typeII monoester, P.D. is packing density in molecules/nm^2

At all sonication times LP were observed. The structure of LP remains the same except for changes in A and β. Ester is forming from solution sonicated longer than 4 hours. A of LP and Ester is smaller compared to that formed in undecanol. This is due to the shorter chain length of decanol, which has one carbon atom less than undecanol. Therefore, a difference of 2.5 Å difference is expected which is well reproduced in the experiment. The general tendency of Ester and LP formation form differently sonicated solutions is the same as for undecanol. A of LP increases as the sonication time increases. Below there is shown a STM image with coexisting LP and Ester structures formed from TMA-decanol solution sonicated for 8 hours.

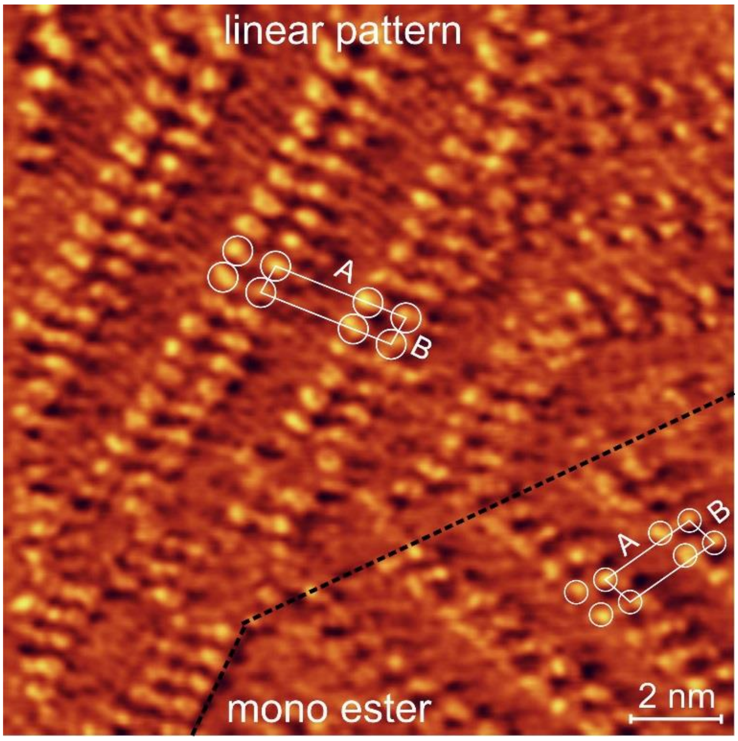

Figure S6: STM constant height image (1.3 V, 1 nA) of adsorption of linear and *Ester* patterns deposited from a solution of TMA in decanol at 8 hours sonication.

a)

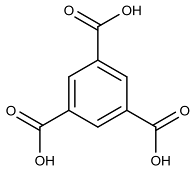
O
OH
O
O
OH
OH

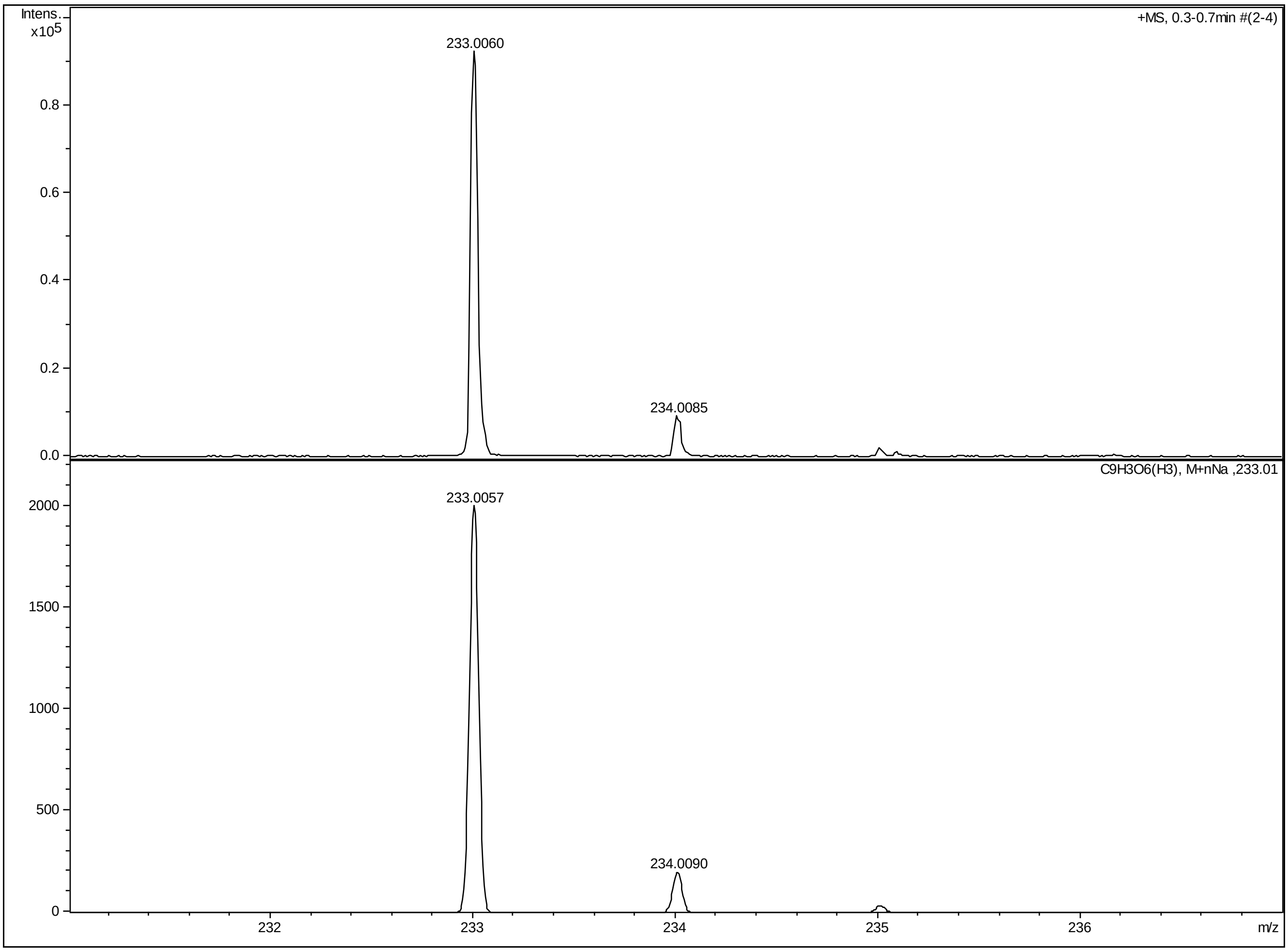
Intens.
x10^5
+MS, 0.3-0.7min #(2-4)
233.0060
234.0085
0.8
0.6
0.4
0.2
0.0
C9H3O6(H3), M+nNa ,233.01
233.0057
234.0090
2000
1500
1000
500
0
232
233
234
235
236
m/z

b)

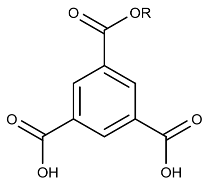
O
OR
O
O
OH
OH

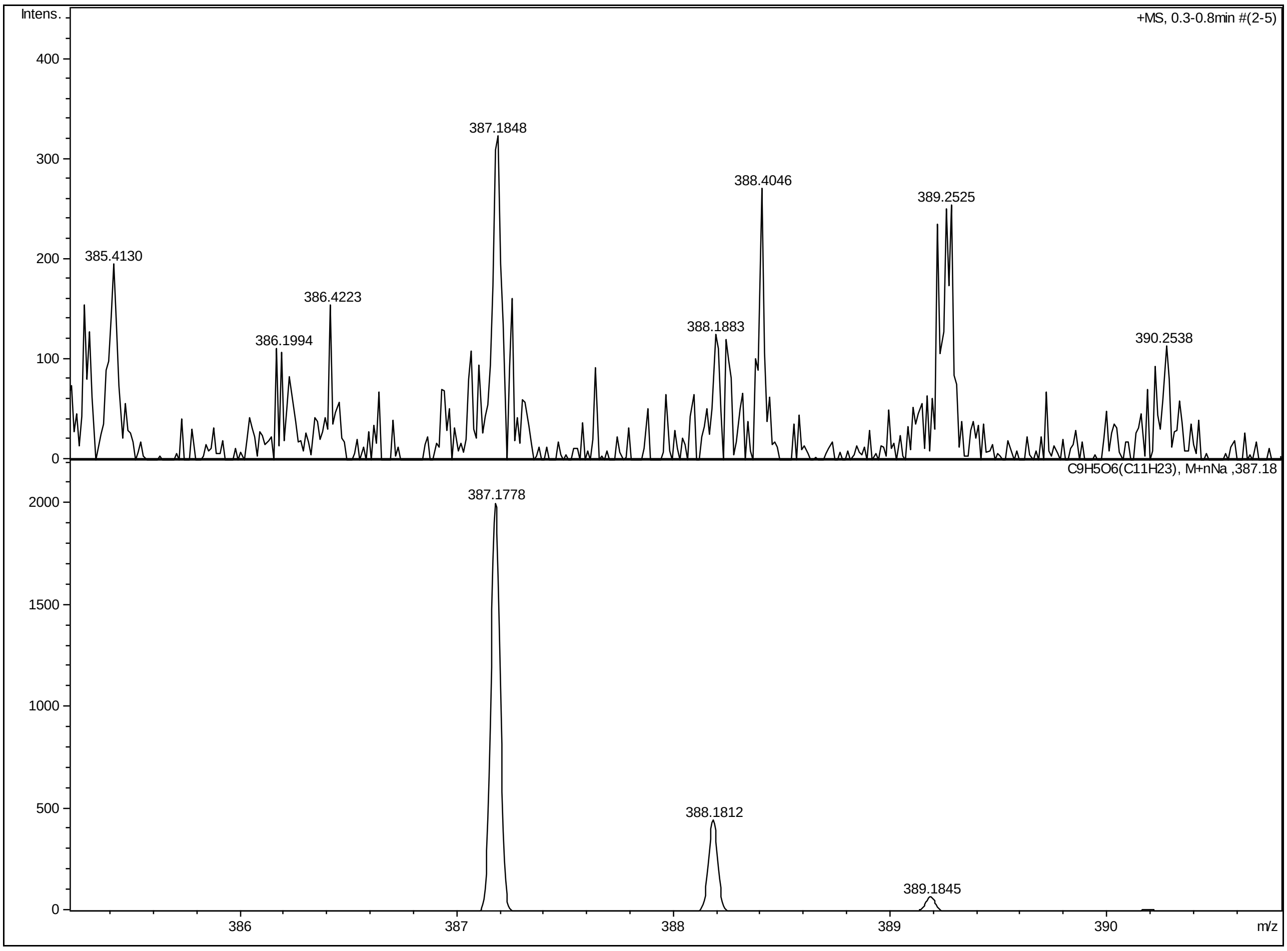
Intens.
+MS, 0.3-0.8min #(2-5)
385.4130
386.1994
386.4223
387.1848
388.1883
388.4046
389.2525
390.2538
C9H5O6(C11H23), M+nNa ,387.18
387.1778
388.1812
389.1845
m/z

c)

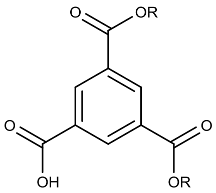
O
OR
O
O
OH
OR

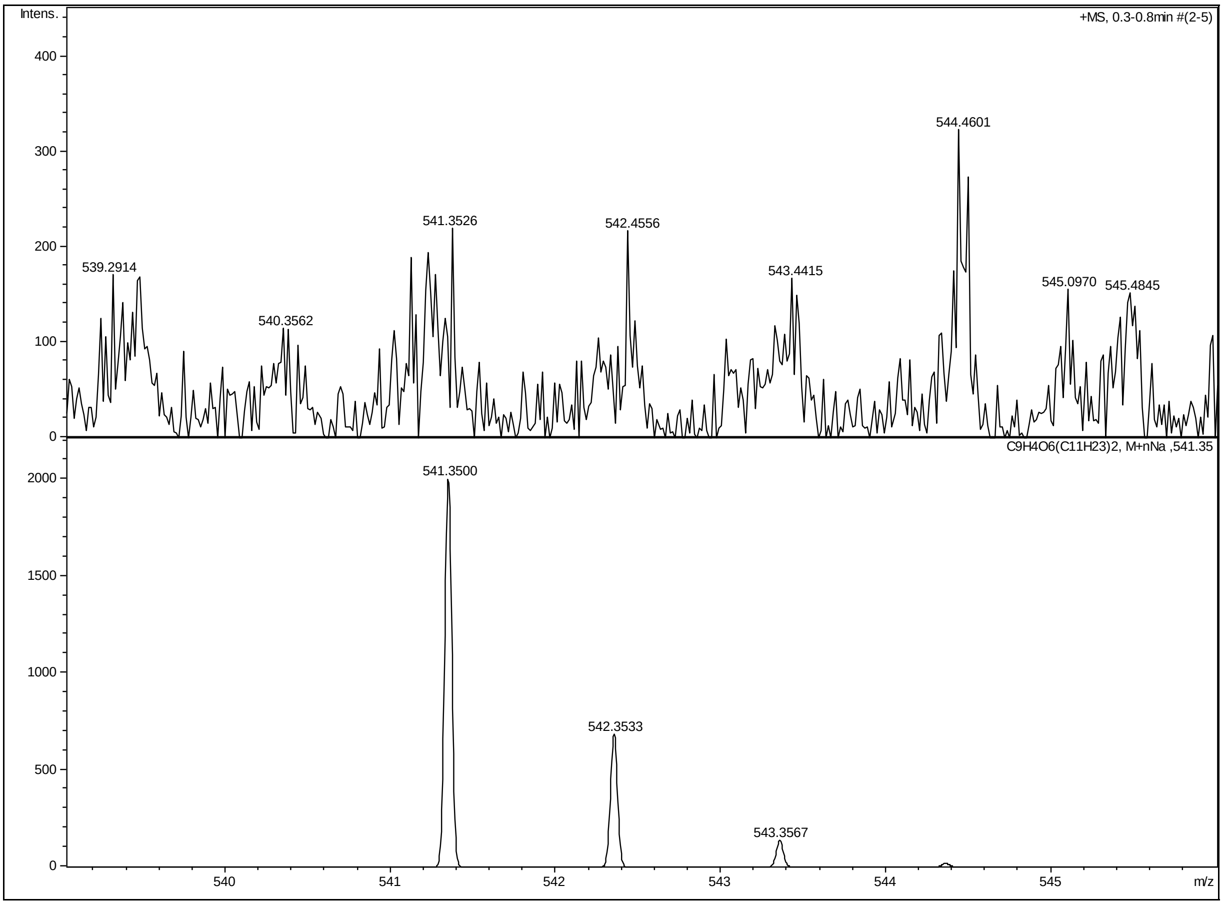
Intens.
+MS, 0.3-0.8min #(2-5)
400
300
200
100
0
544.4601
541.3526
542.4556
539.2914
543.4415
545.0970
545.4845
540.3562
C9H4O6(C11H23)2, M+nNa ,541.35
2000
1500
1000
500
0
541.3500
542.3533
543.3567
540
541
542
543
544
545
m/z

d)

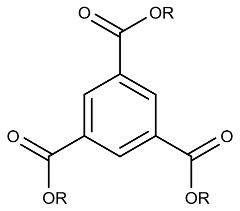

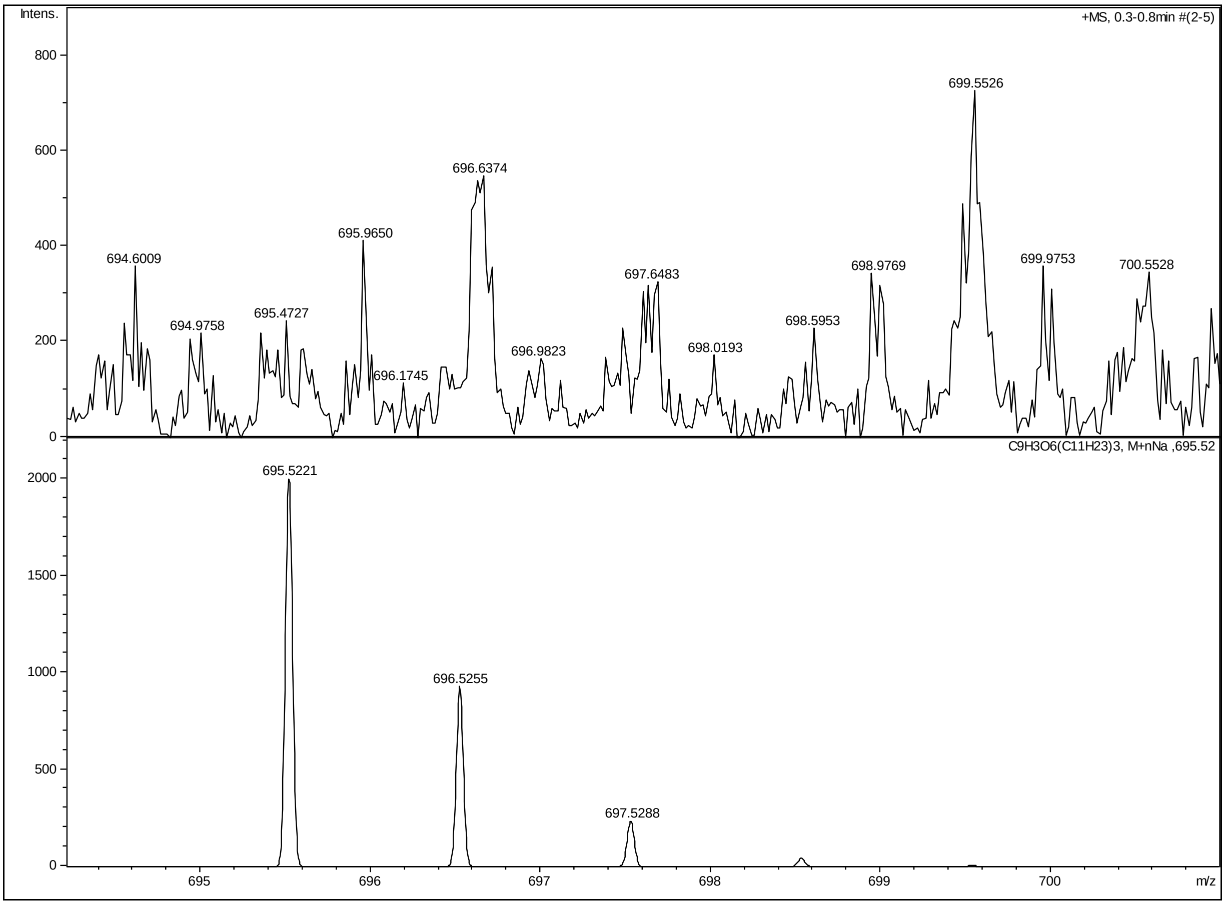

Figure S7: Parts of ESIMS spectrum of the ultrasonicated solution (8 hours) of TMA and undecanol (measured using acetonitrile as solvent; measured (above), calculated (below)) showing the region of the sodium-ion adducts of a) TMA, b) TMA-undecyl monoester, c) TMA undecyldiester and d) TMA undecyltriester (R = $C_{11}H_{23}$). The spectra show clearly that large amounts of the free acid (TMA) are still present (a) and small amounts of the monoester accrued (b). The diester might be also present in extreme low concentration (c), while the triester is not detectable by ESIMS.

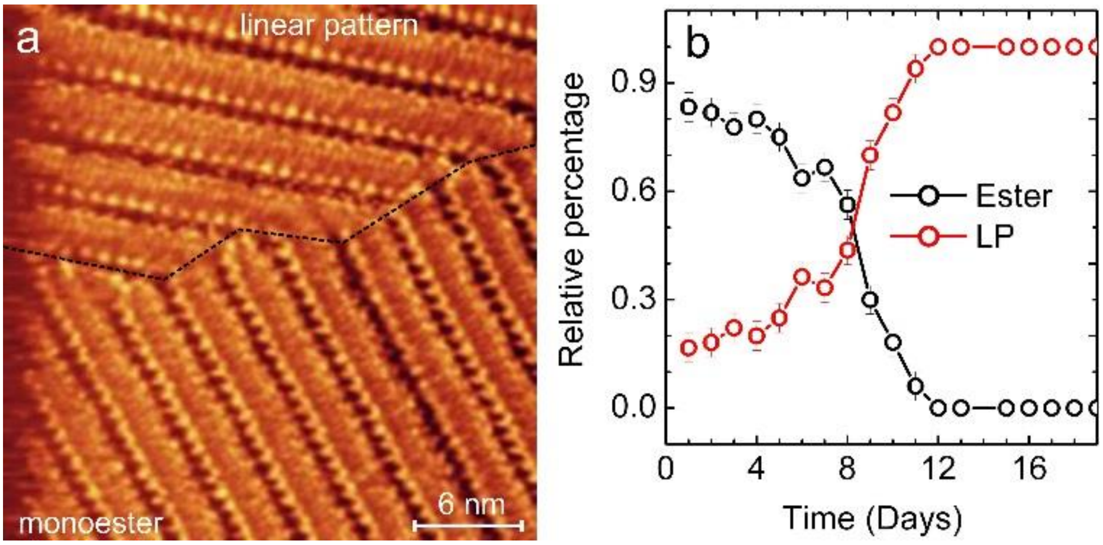

Figure S8: (a) STM constant height image (1.2 V, 1 nA) showing a domain boundary between original linear pattern and monoester structure of TMA undecanol solution sonicated to 8 hours. b) Relative percentage of Ester and linear pattern formed from 8 hours sonicated solution as a function of time (in days). After 12 days no Ester has been found at the interface.

To investigate the extent of esterification at interface we have studied the relative percentage of Ester and linear pattern (LPs) at the interface as a function of time (days) after sonicating solution up to 8 hrs. Several areas (50 × 50 nm^2) with coexisting ester and LP structures have been used to calculate the relative percentage on each day. The figure shows such an example for co-existence of linear pattern and Ester from 8 hours sonicated solutions. Since the difference in the lattice parameter A is clearly distinguishable for LP and Ester pattern, we have used 8 hours sonicated solutions for sampling. Experiments were performed regularly at the same time window (10.00 a.m. to 14.00 p.m.) on each day. The general tendency is shown in the figure. Time zero indicates the first day at which the mixture of TMA and undecanol has been freshly prepared by sonication. Approximately 85% of the area consists of

monoester and the rest is LP. The experiment has been continued and images have been acquired in intervals of one day. Interestingly the percentage of monoester decreases with increasing time. After 12 days the entire surface has been covered with LP. In addition, average A of LP has been decreased systematically with time. At time zero it was 3.9 nm and then reduced to 3.6 nm after 9 days, finally reduced to 3.4 nm after 12 days. That is the LP structure observed after 12 days is very similar to pure LP0.

In our previous study, the polymorphs of TMA in octanoic acid driven by concentration have been observed to be always the same for several months (> 4 months), confirming the long-time stability of the solution [7,8]. That means sonication allows to fabricate a stable saturated solutions of TMA and octanoic acid. Unlike, the concentration driven polymorphs in TMA-octanoic acid mixture, TMA-undecanol mixtures shows a limited repeatability time for concentration driven products. That is TMA and alcohol molecules presumably form a supersaturated solution, which is not stable for longer time. This is possibly due to stronger interaction between undecanol and TMA compared to TMA and alkanoic acid. Sonication allows to increase the solubility of solute in solvent via breaking the solvent-solvent interactions [7,8]. These interactions are re-established within the solvent after given time. That is the concentration of TMA decreases as time evolves and therefore the ester formation at the interface vanishes.

References

1. Ahn, S.; Matzger, A. J. *J. Am. Chem. Soc.* **2012**, *134*, 3208–321.
2. Carola Mende, PhD thesis, TU Chemnitz, Faculty of Natural Sciences, Institute of Chemistry, 2017, to be submitted.
3. Zhao, J.; Khalizov, A.;Zhang, R.;McGraw, R. *J. Phys. Chem. A*, **2009**, *113*, 680.

4. Ha, N. T. N.; Gopakumar, T. G.; Gutzler, R.; Lackinger, M.; Tang, H.; Hietschold, M. *J. Phys. Chem. C* **2010**, *114*, 3531-3536.
5. Nath, K. G.; Ivasenko, O.; Miwa, J. A.; Dang, H.; Wuest, J. D.; Nanci, A.; Perepichka, D. F.; Rosei, F. *J. Am. Chem. Soc.* **2006**, *128*, 4212-4213.
6. Nath, K. G.; Ivasenko, O.; MacLeod, J. M.; Miwa, J. A.; Wuest, J. D.; Nanci, A.; Perepichka, D. F.; Rosei, F. *J. Phys. Chem. C* **2007**, *111*, 16996-17007.
7. Ha, N. T. N.; Gopakumar, T. G.; Hietschold, M. *J. Phys. Chem. C* **2011**, *115*, 21743-21749.
8. Ha, N. T. N.; Gopakumar, T. G.; Hietschold, M. *Surf. Sci.* **2013**, *607*, 68–73.
